# Overrepresentation of transcription factor families in the genesets underlying breast cancer subtypes

**DOI:** 10.1186/1471-2164-13-199

**Published:** 2012-05-22

**Authors:** Himanshu Joshi, Silje H Nord, Arnoldo Frigessi, Anne-Lise Børresen-Dale, Vessela N Kristensen

**Affiliations:** 1 Department of Clinical Molecular Biology and Laboratory Sciences (EpiGen), Division of Medicine, Akershus University Hospital, Lorenskog, Norway; 2 Institute for Clinical Medicine, University in Oslo, Oslo, Norway; 3 Department of Genetics, Institute for Cancer Research Oslo University Hospital, Radiumhospitalet, Norway; 4 Department of Biostatistics, Institute of Basic Medical Sciences, University of Oslo, Blindern, 0317, Oslo, Norway

## Abstract

**Background:**

The human genome contains a large amount of *cis*-regulatory DNA elements responsible for directing both spatial and temporal gene-expression patterns. Previous studies have shown that based on their mRNA expression breast tumors could be divided into five subgroups (Luminal A, Luminal B, Basal, ErbB2^+^ and Normal-like), each with a distinct *molecular portrait*. Whole genome gene expression analysis of independent sets of breast tumors reveals repeatedly the robustness of this classification*.* Furthermore, breast tumors carrying a *TP53* mutation show a distinct gene expression profile, which is in strong association to the distinct *molecular portraits*. The mRNA expression of 552 genes, which varied considerably among the different tumors, but little between two samples of the same tumor, has been shown to be sufficient to separate these tumor subgroups.

**Results:**

We analyzed *in silico* the transcriptional regulation of genes defining the subgroups at 3 different levels: 1. We studied the pathways in which the genes distinguishing the subgroups of breast cancer may be jointly involved including upstream regulators (1^st^ and 2^nd^ level of regulation) as well as downstream targets of these genes. 2. Then we analyzed the promoter areas of these genes (−500 bp tp +100 bp relative to the transcription start site) for canonical transcription binding sites using Genomatix. 3. We looked for the actual expression levels of the identified TF and how they correlate with the overrepresentation of their TF binding sites in the separate groups. We report that promoter composition of the genes that most strongly predict the patient subgroups is distinct. The class-predictive genes showed a clearly different degree of overrepresentation of transcription factor families in their promoter sequences.

**Conclusion:**

The study suggests that transcription factors responsible for the observed expression pattern in breast cancers may lead us to important biological pathways.

## Background

Previous studies have shown that breast tumors can be divided into five subgroups (Luminal A, Luminal B, Normal-like, ErbB2 over-expressing, and Basal-like) based on their mRNA expression patterns [1]. These patterns have been validated in independent datasets representing different laboratories, platforms and different patient cohorts [2]*.* Survival analyses on a sub-cohort of patients with locally advanced breast cancer showed a significant difference in outcome of the patients in the various expression subgroups, with poor prognosis for the ErbB2^+^ and basal-like subtypes [2]. The expression of 552 genes, the *intrinsic gene list,* has been suggested to be sufficient to separate breast carcinomas into the five distinct subgroups. What mechanisms of common regulation make these genes cluster together? We have previously shown that we can separate the patient clusters based only on the promoter composition of single binding sites in the promoters of the genes from the intrinsic gene list [3]. However, regulation of gene expression in eukaryotes is highly complex and depends on sets of TFs rather than individual TFs [4] and in this study we attempt to characterize the overrepresentation of entire TF families. The promoter composition of the genes is one of the major determinants of gene regulation including multiple transcription binding sites that interact with a specific combination of transcription factors (TF). Eukaryotes achieve this diversity by combining a small number of transcription factors whose activities are modulated by diverse sets of conditions [5]. Different functionalities can be conferred on one TF by its association with different co-factors. These factors may act as global TFs that assist their gene-specific partners in their function, and may thus activate or repress transcription depending on the partner motif and the condition [5]. Analyzing transcription network dynamics in yeast, Luscombe et al*.* showed that, in response to diverse stimuli, transcription factors may alter their interaction patterns to varying degree, thereby rewiring the network [6]. While few transcription factors serve as permanent hubs, most of them act transiently during certain conditions. Exogenous processes like environmental responses facilitated fast signal transductions to multiple genes with short regulatory cascades, whereas endogenous processes needed to progress through multiple stages with a complex combination of TFs to fewer target genes [6]. The same TFs may act both in endogenous and exogenous processes. Regulatory hubs targeting disproportionately large numbers of genes and thereby representing the most influential components of a network- have been described. Both Pilpel [5] and Luscombe [6] concluded that precise regulation of a condition cannot arise from the specificity of individual TFs, therefore combinatorial TF usage seems to be the key. The NF-κB family of TFs is an example of transcription regulators that are activated by both intra- and extra-cellular stimuli such as cytokines, oxidant-free radicals, ultraviolet irradiation, and bacterial or viral products [7]. Aberrant NF-κB activity has been implicated in carcinogenesis and in the control of cellular response to anti-cancer agents. Activated NF-κB was detected predominantly in ER-negative breast tumors, and mostly in the ErbB2over-expressing tumor subgroup [8].

## Methods

The *in silico* analysis of the transcriptional regulation of genes defining the subgroups was performed at three different levels: (1) Study of the pathways in which the genes distinguishing the subgroups of breast cancer may be jointly involved including upstream regulators (1^st^ and 2^nd^ level of regulation) as well as downstream targets of these genes. (2) Then we analyzed the promoter areas of these genes (−500 bp tp +100 bp relative to the transcription start site) for canonical transcription binding sites using Genomatix. (3) We looked for the actual expression levels of the identified TF and how they correlate with the overrepresentation of their TF binding sites in the separate groups.

### Selection of genes

The expression of 552 genes, the *intrinsic gene list*, which has been suggested to be sufficient to separate breast carcinomas into the five distinct subgroups defined in [1] and [2,9] was used for the pathway analysis in this study (referred to as *full* list). A subset consisting of 197 genes [10] that best represented the classification scheme in breast cancer (referred as *top* list) were selected from the *intrinsic list*, and used in the promoter analysis part (Additional file 1: Table S1)*.*

### Pathway analysis

Pathway analysis was performed using Pathway Studio [11] from Ariadne Genetics. Two network prediction algorithms were used that allow to discover the patterns of gene expression inherent in the experimental data: Pearson Correlation and Auto Net Finder network prediction algorithm. Pathway Studio’s text mining tools were applied to extract biological associations by mining PubMed to build pathways from extracted facts using data from recent publications and public and commercial databases such as KEGG, BIND, GO, and the PathArt database of curated signaling and disease pathways. The algorithm for building Correlation Network in Pathway Studio is based on Pearson Correlation. Genes with similar expression profiles are connected with edges indicating the significance of the correlation. The group of tightly correlated genes form cluster in the correlation network. The algorithm can be used for clustering genes according to their expression profiles across multiple samples. The tool calculates correlation coefficients between all pairs of gene expression profiles measured in the experiment and outputs clusters of highly correlated genes. Identified gene clusters can be further validated and analyzed using relations from the database that have been extracted from the literature by Ariadne Genetics. Auto Net Finder is a network estimation system that combines hierarchical clustering and Graphical Gaussian Modeling and is used for distinguishing direct and indirect relationship among variables. Bibliosphere pathways (release 7.1) [12] (http://www.genomatix.de, Genomatix Software GmbH) was used for extracting the associations between gene, transcription factor and proteins corresponding with the genesets defining each molecular subtype of breast cancer. Genomatix Bibliosphere is a knowledge database consisting of manually curated co-cited genes in PubMed, which additionally provides information about the presence of TFBS in their promoters, using *in silico* tool- MatInspector, interactions and associated pathways from Molecular Interactions database-NetPro and BioCyc, respectively.

### Analysis of overrepresentation of TFBS families in the promoter sequences

We extracted the putative regulatory promoter regions from 500 bp upstream to 100 bp downstream of RefSeq promoters of the subtype-associated genes. Further analysis was based on the hypothesis that overrepresentation of potential transcription factor binding site (TFBS) motifs in a set of co-expressed gene promoters may indicate regulatory relationship. In order to emphasize the functional representation of TFBS motifs overrepresented in a set of promoters, we used the TFBS matrix family concept. TFBS matrix families are defined as groups of TFBS weight matrices corresponding to the same or functionally similar transcription factors. For any given TF, there could be multiple matrices described by different independent sources, leading to multiple matches for similar position or shifting of matches by a few base pairs. By using the functional domain clustering based on di/tri/tetra-nucleotide occurrence and additionally function-based subgrouping, TFBS matrices can be grouped according to their functional similarity, known as TFBS families [13]. Thus members sharing same TFBS family are expected to have functional similarity in addition to binding domain similarity. For estimation of over-representation of each TFBS family, first occurrences of its corresponding TFBS motifs within a set of subtype-specific promoter sequences was obtained. Then relative occurrence of each TFBS family was estimated by comparing this observed occurrence to the rate of occurrence of the same TFBS matrix family in an equal base-pair long reference background sequences from human promoter. Overrepresentations of a motif is measured by two different methods:

1. In terms of fold factor of overrepresentation compared to the backgroundFold factor of TFBS overrepresentation was calculated by a formula as mentioned below:

(1) $$r(X)=\frac{n_{\mathrm{obs}}(X)}{n_{\exp}(X)}$$

Where, *r*(*X*) = fold factor of overrepresentation of a TFBS family, *Xn*_*obs*_ (*X*) = observed number of hits of *X* in a given set of promoter sequences*n*_*exp*_ (*X*) = expected number of hits of X in an equally sized sample from genomic promoter background sequences

2. As *z*-scores that provide a measure of the distance of sample from the reference population mean. Here sample refers to the number of observed hits of any particular TFBS in a given input set of sequences and reference refers to the number of hits of the same TFBS in equally sized human genomic promoter sequence population.

(2) $$z(X)=\frac{n_{\mathrm{obs}}(X)-n_{\exp}(X)-0.5}{S(X)}$$

*z*(*X*) is a *z*-score of overrepresentation of a transcription factor binding site family (*X*);

*n*_*obs*_ (*X*) is a number of observed hits of *X* in an input promoter sequences;

*n*_*exp*_ (*X*) is expected number of hits of *X* in an equally sized sample sequences in human genomic promoter background;

*S*(*X*) is a population standard deviation of number of hits of *X*

We used Genomatix RegionMiner tool (Genomatix Software GmbH, http://www.genomatix.de) in order to evaluate the degree of TFBS family overrepresentation. The histogram of *z*-scores of each TFBS motif families in each subtype-specific promoter sequences is shown in the Additional file 2: Figure S1*.* Histograms like this indicate that choosing the cut-off level of 2.0 allows identifying TFBS families that are overrepresented. However, *z*-score cut-off level of 2.0 does not provide a precise measure of significance, because of the disparity of sample size between sample and reference. Due to the copyright and technical limitations in accessing the Transfac database, further statistical testing of over-representation could not be performed within that tool.

Under-representations or absence of TFBS family motifs in sub-type specific genes may occur due to a fewer number of subtype-representative genes and subsequently a smaller number of promoter sequences used for any particular subtype. This can be a source of false positivity. Therefore we have not taken into account the under-representations of TFBS family motifs in this analysis.

### Principal component analysis to identify TFBS with maximum variance between subtypes

Principal component analysis (PCA) [14] was performed for ranking the TFBS families with respect to the variance of fold-factor overrepresentation contributed by them between five subtypes. We prepared a matrix of TFBS fold-factors for subtypes, with subtypes as columns and TFBS families as rows. We performed PCA on this matrix using the *princomp* function of *Matlab*. Subtracting each data point from the column mean represents a center of this matrix. Hotelling’s *T*^*2*^ statistic was used as a measure of multivariate distance of each TFBS family from the center of the TFBS fold-factor matrix as described in http://www.mathworks.com/help/toolbox/stats/princomp.html.

### Gene expression data

We used a subset of the samples (*n* = 114) from previously published [15] mRNA expression data [GEO dataset #GSE19783]. Subtypes were predicted by using the *PAM50*[16].

### mRNA expression of the studied TF

Transcription factor families with overrepresentation *z*-score >2.0 were mapped to their corresponding probes in the mRNA expressions dataset. By applying multiclass SAM, we extracted 120 TF genes with significantly different (at the FDR <0.1) expression between the five subtypes. Pearson’s correlation between the subtype-specific geometric mean expression of this subset of transcription factor genes and fold overrepresentation was computed. The justification of using geometric mean instead of arithmetic mean is that typically mRNA expression values are log-normally distributed.

## Results and discussion

### Pathway analysis of the genes that define the five breast cancer subgroups

Using Pathway Studio from Ariadne Genetics, we studied the direct interactions between the genes with distinguished gene expression pattern in the breast cancer subgroups as described in *Materials and Methods, selection of genes*. Most profound direct interactions were observed for the genes defining the luminal A group with protein-protein interactions between *XBP1* and *ESR1* and *CCND1* (Additional file 3: Figure S2)*.* Trefoil *(TFF3*) has been functionally coupled to *CCND1* through angiotensin receptor 1 (*AGTR1*). Angiotensin II is converted from its precursor by angiotensin I-converting enzyme (ACE) and has been shown to mediate growth in breast cancer cell lines via ligand-induced activity through the angiotensin II type 1 receptor (*AGTR1*). We also searched for upstream regulators as well as downstream targets of these genes. Downstream targets could be observed centered at the ESR1, MYC, NFKB1, GATA3, CCND1, TP53 and MSX2/FOXC1 (Additional file 4: Figure S3).

A somewhat less organized pathway structure is observed in the luminal B subclass. The ESR1 node was not observable and the TP53 network was more sparse with fewer partner genes. Novel nodes were centered at NRG1, GSTP1 and CUL1 (Additional file 5: Figure S4)*,* CUL1 has homology to yeast Cdc53, which is part of a complex known as SCF that mediates the ubiquitin-dependent degradation of G1 cycles and cyclin-dependent kinase inhibitors, while NRG1 contains a domain related to the epidermal growth factor family of ligands and can act as receptor agonists. The direct interactions between genes highly expressed in Luminal B subtype were observed between *GSTP1* and *CDK2AP1*, *S100A10* and *S100A11* and *PPP1R13B* and *TP53BP2*. The latter protein interacts with *TP53* to specifically enhance p53-induced apoptosis but not cell cycle arrest.

Four distinct regulatory nodes were observed in the ERBB2 group: around the ERBB2 itself, TP53, NFKB1 and CTNNB1 (cadherin-associated protein, beta 1) (Additional file 6: Figure S5)**.** NFkB-p65 was shown to repress β-catenin-activated transcription of cyclin D1 [17]. Moreover, a direct interaction is established between *ERBB2* and *GRB7* (Additional file 3: Figure S2)*.* The solution structure of the Grb7-SH2/erbB2 peptide complex was described and suggested to be involved in cell signaling pathways that promote the formation of metastases and inflammatory responses. *PPARBP*, which is co-amplified with ERBB2, has in early studies been suggested to play a role in mammary epithelial differentiation and in breast carcinogenesis by its ability to function as *ESR1* coactivator. It was shown to contain a typical CCAT box and multiple cis-elements such as C/EBPbeta, YY1, c-ETS-1, AP1, AP2, and NFkappaB binding sites. The 4 different regulatory nodes are connected by *FLOT2,* the human epidermal surface antigen involved in epidermal cell adhesion. *NFKB1* was present in the network for the Basal group, where also the FOX family, a whole family of cyclins and CDK2, and CDK6 and isoforms of protein kinase (*RPS6K*) were present (Additional file 7: Figure S6). Interestingly, a large number of connections lead to *GJA1* (Cap junction protein, alpha, also known as connexin 43). Other distinct nodes around TP53 are those connecting to KRT5, MAPK signalling, E2F1 and NCL. NCL, Nucleolin, one of the most abundant nucleolar proteins, has been recently shown to be involved in the reprogramming of somatic cells for derivation of either embryonic stem (ES) cells, by somatic cell nuclear transfer (SCNT), or ES-like cells, by induced pluripotent stem (iPS) cell procedure. Nucleolar proteins are proposed to be the markers of activation of embryonic genes [18] and provide mechanism for nucleolar control of progression of cell cycle in stem cells and cancer cells [19]. TP53 was a central node in the regulatory network of the normal-like subgroup, surrounded by JUN, ACSS2, ACSL1, KRT13, PIK3R1 and other nodes some representing glycolysis, energy metabolism, pyruvate metabolism and metabolism of *carbohydrate* (Additional file 8: Figure S7).

**Figure 1:**
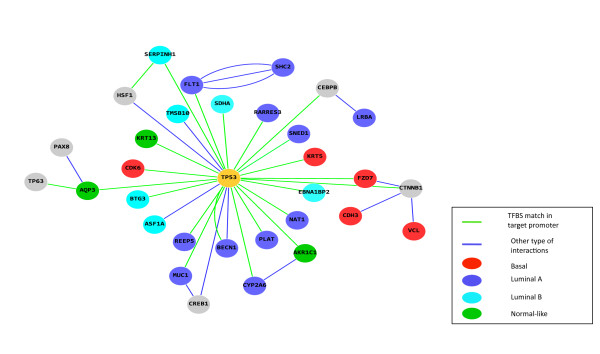
**Predicted functional relationship of TP53 in different molecular subtypes of breast cancer.** Figure shows predicted interactions of genes or proteins with TP53. Source: *Bibliosphere pathway database*. (*green edges*: TF motif match found in target promoter of target genes; genes associated with basal subtype are shown as *red* nodes, ones with luminal A in *blue*, luminal B in *cyan* and normal-like as *green* nodes.)

Noteworthy, a TP53 network node was observed in each of the studied expression subclasses shown here (Additional file 4: Figure S3, Additional file 8: Figures S7)*.* It is of interest to note that in every case TP53 was a hub in a somewhat different neighborhood. While in the basal subtype TP53 was connected to CDK6, a cyclin-dependent protein kinase (CDKs) that regulate major cell cycle transitions and CDH3, cadherin 3, as well as FZD7 and KRT5, in the luminal A tumors one could observe detoxifying enzymes such as NAT1, CYP2A6 as well as the retinoic acid receptor RARRES3 in the TP53 hub (Figure 1)***.***

### Over-representation of specific transcription factor binding sites in the promoter of the genes that distinguish the subtypes

**Figure 2:**
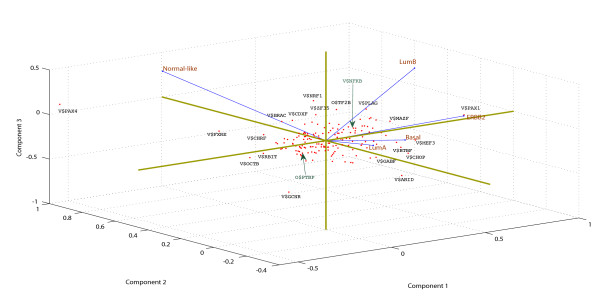
**PCA plot of overrepresentated TFBS matrix families.** PCA plot shown in terms of fold factor of overrepresentation in each subtype compared to the reference genomic promoters background. Blue lines represent the Eigen vectors, the direction and length of which indicates how each subtype variable contributes to the principal components in the plot. TFBS matrix families with maximum distance from the centroid are labeled on the plot.

The correlation matrix of TFBS fold-overrepresentation vectors for the five subtypes shows positive correlation in terms of potential TFBS family overrepresentation between 1. ERBB2+ and basal subtypes (0.27); 2. Luminal B and ERBB2+ (0.16); 3. Luminal A and luminal B (0.11). In order to visualize the differential TFBS overrepresentation, we performed the principal component analysis (PCA). PCA plot (Figure 2) displays the significant differences between the subtypes in terms of fold-factor of motif frequencies observed in promoter sequences of subtype-associated gene promoters compared to their corresponding normal frequencies in genomic promoter sequences. Distances between points representing the TFBS matrix families are the multivariate distances of fold-factor overrepresentation of each TFBS family in each of the subtype. This indicates that the shorter the distance, the greater similarity in fold-overrepresentation of that particular TFBS family in given subtypes. More than 60% and 76% of cumulative variance is captured by first two components and first three principal components, respectively. The top ten ranking TFBS families in distance from center and some of the functionally significant TFBS families are specifically labeled in the PCA plot. Biplots of first and second principal components show differentially overrepresentated TFBS families between the normal-like and rest of the subtypes. Biplot of second and third principal components shows TFBS family overrepresentations in luminal B. Differential TFBS family representations between ERBB2+ and basal groups cannot be seen in biplots of first three principal components, but can be visualized in a biplot of first and fourth principal components. In the first principal component, V$BTBF, V$PAX1, V$PAX4 and V$TCFF are the major contributors of variance, where as V$PAX4, V$GUCE, V$ARID are the major contributors of variance in the second principal component.

**Figure 3:**
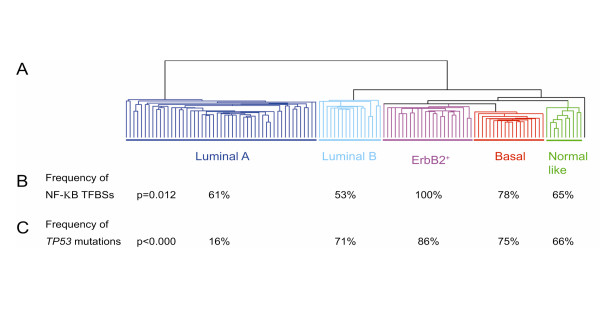
**Subtypes with relevance to NF-κB binding sites and TP53 mutations.A.** The five subtypes shown by hierarchical clustering using the “intrinsic” gene set. Dendrogram shows the clustering of the tumors into five subgroups. Branches are color-coded. **B.** Frequency of NF-κB binding sites in the 5 subgroups; and **C. **Frequency of *TP53* mutations.

Several of the gene clusters shared *cis*-elements that were present in more than 90% of the promoters. For the top six genes that classify the ErbB2+ over-expressing cluster, four TFBSs were found to be present in 100% of the promoters. These were NOLF (Neuron-specific-olfactory), ETSF (E26 Transformation-Specific factor 1), STAT (the Signal Transducers and Activator of Transcription protein) and NF-κB (Nuclear Factor κappa Beta) (Additional file 9: Table S2)*.* NF-κB is the family of nuclear factor kappa beta of transcription factors. NF-κB has been shown to promote cell proliferation, to suppress apoptosis, to promote cell migration, and suppress differentiation [7]. NF-κB binding sites were found significantly over-represented in the promoters that best classify the ErbB2^+^ subgroup compared to the other 4 subgroups (Additional file 9: Table S2; Figure 3B) and 78% of the 27 genes expressed in the basal-like subgroup had also NF-κB binding site in the promoter. This was in marked contrast compared to the promoter composition of the normal-like and luminal subgroups (Figure 3B)*.* The presence of NF-κB binding sites in the genes from the ERBB2 and basal groups is in concordance with the pathway analysis performed on the downstream genes (see above). The *cis*-elements PAX1, PAX9 (The paired box gene 5), MAZF (*myc*-associated zinc finger) and EGRF (epidermal growth factor receptor) were overrepresented in the genes that are over-expressed in the Luminal B subgroup (Additional file 9: Table S2). While the PAX superfamily is involved in a multitude of developmental processes and is required for initiating B cell lineage and maintaining neural development and spermatogenesis, the MAZF is a common transcription factor and might play a more general role. The major distinction between the luminal A and B, both consisting of ER positive tumors, is the presence of a strong proliferations cluster in the luminal B subtype. Noteworthy, binding sites for growth factors and their receptors like EGRF are over-represented in the promoters of the genes that define the luminal B subgroup and were overrepresented in the pathway analysis as well *(see above)*. EGRF is not only a receptor for EGF (Epidermal growth-factor), but also for other members of the EGF family and it is involved in the control of cell growth and differentiation. For the geneset of the normal-like subgroup, we observed overrepresentation of NRF1 family of TFBS (Additional file 9: Table S2).

### Presence of promoter modules in genes that define the ErbB2+ subgroup

**Figure 4:**
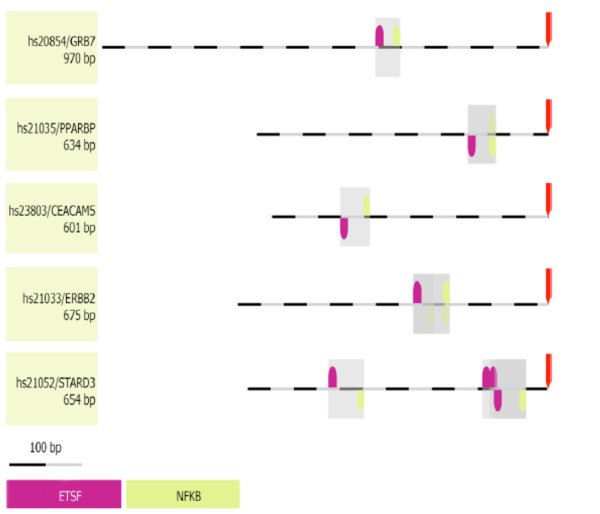
**Common Framework in the ErbB2**^**+**^**subgroup.** The common framework consisting of NF-κB and Ets found in the 6 cluster defining genes of the ErbB2+ over-expressing subgroup. Distance to next element is between 29 and 79 bp (ETSF). Directions (up/down) of the elements indicate presence of hits on sense or antisense strands respectively.

The specificity of promoter-controlled gene regulation may depend on the relative organization of the elements within the promoter rather than solely on individual elements [20-22]. Genes expressed in the same functional context do often share promoter modules [20,21]. The binding elements are often occupied differently in different tissues, and these differences can be used to derive all type-specific sub-modules *in silico*. A promoter module may be defined as an organized group of regulatory elements where both order and distance should be considered. Genes expressed in the same functional context do often share promoter modules [20,21]. For the six best genes of the ErbB2+ over-expressing cluster, a common framework consisting of NF-κB and ETS1 transcription factor binding sites was found (Figure 4)*.* The ETS are fundamentally important TFs with roles in cell development, cell differentiation, cell proliferation, apoptosis and tissue remodeling (reviewed [23]). The family is characterized by an evolutionarily conserved DNA-binding domain that regulates expression by binding to a purine-rich core sequence in cooperation with other TFs. Most of the proteins in the ETS family are downstream nuclear targets of *ras*-MAP kinase signaling, and the deregulation of ETS genes results in the malignant transformation of cells [24] It has previously been reported that mutant TP53 required ETS1 to synergistically activate the expression of *ABCB1*. ETS1 was shown to interact exclusively with mutant TP53 in vivo, but not with wild-type TP53 [25]. High levels of ETS1 expression were associated with poorer prognosis [26]. The presence of a promoter module constituting of NF-κB and ETS has been reported previously in genes co-regulated in mitogen-stimulated T-cells [27]. Interactions between members of the ETS family and NF-κB have been described previously. ETS1 induces IKKα expression. IKKα is a kinase that marks the NF-κB inhibitor IκB for degradation, and active NF-κB is translocated to the nucleus. ETS1-mediated activation of IKKα is negatively regulated by TP53 binding to ETS1. TP53 physically interacts with ETS1 and specifically inhibits ETS1 induced IKKα promoter activity. Loss of TP53-mediated control over ETS1 dependent transactivation of IKKα may represent a novel pathway for the constitutive activation of NF-κB mediated gene expression and therapy resistance in cancer cells [28] TP53 is therefore an ETS1 and ETS2 target gene [29]. NF-κB controls a broad spectrum of genes by a variety of mechanisms in response to diverse environmental changes. NF-κB may be a universal regulator, while ETS could reflect cell-type or stimulation specific differences since ETS binding sites were detected in a fraction of the NF-κB controlled genes.

### Over-representation of TP53 mutations in the tumors that belong to the ErbB2^+^ and basal-like subgroups

In human breast tumors, the two tumor subgroups exhibiting the most prominent activation of putative NF-κB target genes (ErbB2^+^ and Basal-like) also harbored the highest frequency of p53 mutations. 86% of the patients in the ErbB2^+^ subgroup had *TP53* mutations in their tumors and all the genes that are abnormally expressed in this tumor type have NF-κB binding sites in their promoter (Figure 3C)*.* There is an evidence that NF-κB can regulate *TP53* expression and that NF-κB is required for TP53-dependent cell death [30]. In turn, TP53 activates NF-κB through the RAF/MEK1/p90 pathway [30]. The TP53 protein interacts with NF-κB and enhances its transcriptional activity and its anti-apoptotic efficacy. Over-expression of ErbB2 is known to induce the classical NF-κB pathway [31,32]. The estrogen receptor (ER) can bind physically to NF-κB to inhibit its DNA binding functions, hitherto repressing gene expression [33]. Therefore the NF-κB pathway was shown to be a major stroma-tumor signaling mediator in ER negative tumors with over-expression of ErbB2 [8]. NF-κB signaling has been associated with doxorubicin resistance, and agents blocking NF-κB function have been proven beneficial in the treatment of tumors in combination with standard anti-cancer therapies [34].

### Over-represented transcription factor families within the promoter sequences

**Figure 5:**
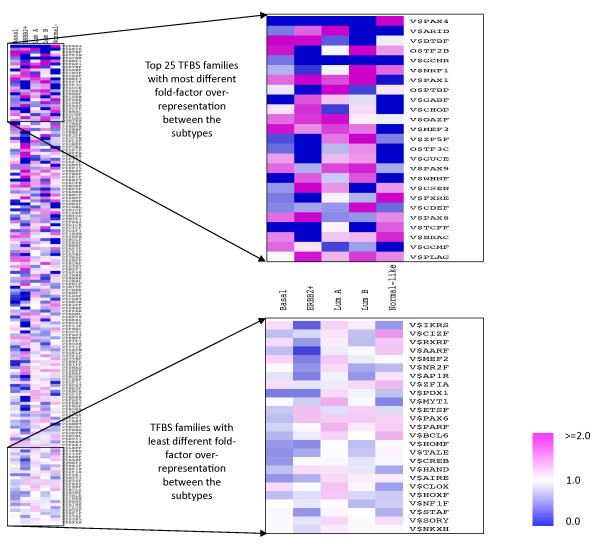
**Heatmap of fold overrepresentations for the TFBS families ranked according to their distance from center.** Out of all, 25 TFBS matrix families top ranked according to their distance from the centroid of fold-overrepresentation matrix, are highlighted

We observed the over-representation of V$BTBF (*kaiso*), V$OAZF and V$PAX8 in basal and ERBB2+ tumor associated gene promoters (Figure 5, Additional file 10: Table S3). *Kaiso* group of transcription factors are known to show nuclear accumulation during active mitosis [35] and their over-representation indicates potential functional role in these two subtypes showing aggressive tumor progression and high cell proliferation. PAX8 activity has also been observed in metastatic renal tumors [36]. Precise role of PAX8 and OAZF groups of transcription factors is yet unknown in breast cancers. ERBB2+ gene promoters also show over-representation of V$NFKB, Pleomorphic adenoma gene associated V$PLAG and *ras*-responsive element binding protein associated V$RREB families of TFBS. Activity of NFKappa B is already discussed in the earlier section. RREB1 activity plays a role in TP53 mediated apoptosis [37] that gets perturbed in absence of functional TP53, which is a common phenomenon in ERBB2+ tumors. Both luminal groups involve over-representation of PAX subgroup 1 member TFBS’s- V$PAX1, V$PAX9 and V$ZF5F families. PAX9 activity is known to be a marker of better prognosis. Overrepresentation of V$P53F, V$HOXF, V$CLOX, V$PARF and V$GATA was observed specifically in luminal A group in which estrogen receptor signaling is a predominant characteristic. The transcription factors corresponding to V$PARF group (PAR bZIP TFs) are mediators in oxidative stress-induced apoptosis [38]. In the luminal B group of promoters, we observed over-representation of V$EGRF, V$CTCF and V$EKLF etc. Egr-1 which corresponds to the V$EGRF family is known to be associated with cell cycle entry in response to growth stimuli [39]. We also observed significant over-representation of V$NRF1 in both normal-like and luminal B group of promoters. NRF-1 transcription factor is an oxidant-sensitive transcription factor, usually found in ER positive breast cancers [40] and is shown to be associated with higher tumor grade [41].

By using the Wilcoxon rank sum test, we observed significantly elevated mRNA expressions of *ESR1* and *PGR* in Luminal A or Luminal samples compared to the basal ones (p < 1.0e-6), with non-significant differences in *ERBB2* expressions. As expected *ERBB2* was significantly upregulated in ERBB2+ tumors along with downregulated *ESR1* and *PGR*, compared to the rest (p < 1.0e-4). Regulation by many transcription factors shown overrepresented here in ER + ve or ER-ve subtypes is not well characterized in context of estrogen and progesterone receptor activity. However, overrepresentation of some of the TFBS, such as GATA, BTBF, NF Kappa B – appear to be consistent with prevailing knowledge about the subtypes and their ER/PR or Her2 status.

Thus functions of the TF genes corresponding to the over-represented TFBS families hint the predominant characteristics of the subtypes. Findings from the above *in silico* analysis will be further validated in reporter studies and ChIP analyses. The approach of identifying overrepresented TFBS in a set of coordinately expressed genes under a particular disease class or condition can improve the specificity and noise tolerance. [42]. However, its main limitation is that it does not account for the role of local chromatin environment constituted by structural properties, epigenetic modification etc. The local chromatin environment can offer condition-specific functionality to the existing TFBSs in a set of promoters. 

Promoter sequences extending from 500 bp upstream to 100 bp downstream relative to TSS typically contain core promoter elements, CpG islands, downstream promoter element and other components of transcriptional machinery. Besides, this region has been demonstrated to have high density of positional as well as comparative TFBS [43], many of which are typically location sensitive. Thus limiting the analysis to this proximal promoter region, rather than analyzing the broader region (i.e. -1000 bp to +500 bp relative to the TSS) – could reduce false positives in TFBS overrepresentation. However, by that very limitation we may omit important information about second alternative promoters and distant control loci, which are therefore outside the scope of this analysis.

### Correlation between actual abundance of TFs and frequency of their BS in the genes defining the clusters

**Figure 6:**
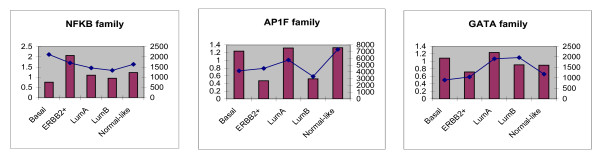
**Correlation between TF overrepresentation and corresponding TF gene in each subtype.** Correlation between geometric mean of TF gene expressions in each subtype (shown as red bars) and their corresponding TF matrix family overrepresented in subtype-specific promoter sequences (shown as blue points) is plotted

Some of the TFBS family overrepresentations were positively correlated with the geometric means of subtype-specific mRNA expressions of their corresponding TF genes. (Shown in Figure 6, Additional file 11: Table S4). The rationale underlying the use of geometric mean is that gene expression intensity values follow lognormal distribution.

Biological uncertainty in a correlation between the abundance of TFs and frequency of their BS might be attributed to several factors. The most common and obvious reason could be mutant or copy number altered TF. Moreover, here we have not accounted for the expressions of downstream targets of the TFs. It is noteworthy that mutations (point mutation and copy number alteration) in TFs can also have an impact on the level of expression of the downstream genes. For instance, a mutant TP53, which is still highly expressed, may not recognize the original binding sites anymore, leading to a drop in the expression of the target genes.

## Conclusion

Here we report that the promoter composition of the genes that strongly predict the patient subgroups is distinct. The gene classes showed a clear separation when based solely on their promoter composition. This finding suggests that studying those transcription factors associated to the observed expression pattern in breast cancers may lead us to important biological pathways responsible for the regulation of gene expression in breast cancer.

## Abbreviations

TF, transcription factor; TFBS, transcription factor binding site; PCA, principal component analysis; ER, estrogen receptor; PGR, progesterone receptor; Her2, Human Epidermal Growth Factor Receptor 2.

## Competing interests

The authors declare that they have no competing interests.

## Authors’ contributions

VNK conceived and designed the study and helped to draft the manuscript. AF provided statistical expertise into the methods used in this study. HJ performed TFBS overrepresentation analysis, wrote the corresponding sections of manuscript, prepared figures and tables and revised the manuscript. SHN participated in pathway analysis using Pathway Studio tool and performed promoter module analysis using MatInspector. VNK and AF approved the final manuscript. All authors read and approved the final manuscript.

## Authors’ information

HJ is a fellow of the Health Authority of South-East Norway. SHN is a fellow of the Norwegian cancer society (Den Norske Kreftforening).

## Supplementary Material

Additional file 1 **Table S1. Subtype-specific gene list.** Table shows the 197 subtype-specific best discriminatory genes, which is a subset of the intrinsic gene-list. Click here for file

Additional file 2 **Figure S1. Histogram of z-scores of overrepresentation.** Histogram of TFBS matrix family overrepresentation observed in subtype-specific promoters compared to the reference genomic promoter background shown as z-scores. Click here for file

Additional file 3 **Figure S2. Direct interactions between genes defining subtypes.** Subtype-relevant key driver interactions for Luminal A, B and ERBB2+ subtypes. Click here for file

Additional file 4 **Figure S3. Protein-protein interactions and TF interactions associated with Luminal A subtype.** Network shown here is based on the luminal A specific genelist. Click here for file

Additional file 5 **Figure S4. Protein-protein interactions and TF interactions associated with Luminal B subtype.** Network shown here is based on the luminal B specific genelist. Click here for file

Additional file 6 **Figure S5. Protein-protein interactions and TF interactions associated with ERBB2+ subtype.** Network shown here is based on the ERBB2+ subtype-specific genelist. Click here for file

Additional file 7 **Figure S6. Protein-protein interactions and TF interactions associated with basal subtype.** Network shown here is based on the basal subtype-specific genelist. Click here for file

Additional file 8 **Figure S7. Protein-protein interactions and TF interactions associated with normal-like subtype.** Network shown here is based on the normal-like subtype-specific genelist. Click here for file

Additional file 9 **Table S2. TFBS overrepresentation in subtypes-specific gene promoters.** List of significantly over-represented transcription factor binding site families in subtypes of breast cancers at the cut-off level of z- score > =2.0. Click here for file

Additional file 10 **Table S3. Over-representation of potential TFBS in subtype-specific promoter sequences.** Table shows the fold over-representation of potential transcriptional factor hits (represented as TFBS families) in subtype- specific gene promoter sequences. Click here for file

Additional file 11 **Table S4. Correlation between TFBS overrepresentation and mRNA expression of corresponding TF genes.** Table displays the Pearson’s correlation between the geometric mean of expression values of transcription factor genes in subtypes and fold overrepresentation of corresponding TFBS families. Click here for file

## References

[B1] Perou CM Sørlie T Eisen MB Van De Rijn M Jeffrey SS Rees CA Pollack JR Ross DT Johnsen H Akslen LA Fluge O Pergamenschikov A Williams C Zhu SX Lønning PE Børresen-Dale AL Brown PO Botstein D Molecular portraits of human breast tumours Nature 2000 406 747 752 10.1038/35021093 10963602

[B2] Sørlie T Tibshirani R Parker J Hastie T Marron JS Nobel A Deng S Johnsen H Pesich R Geisler S Demeter J Perou CM Lønning PE Brown PO Børresen-Dale A-L Botstein D Repeated observation of breast tumor subtypes in independent gene expression data sets Proc Natl Acad Sci USA 2003 100 8418 8423 10.1073/pnas.0932692100 12829800 PMC166244

[B3] Tongbai R Idelman G Nordgard SH Cui W Jacobs JL Haggerty CM Chanock SJ Børresen-Dale A-L Livingston G Shaunessy P Chiang C-H Kristensen VN Bilke S Gardner K Transcriptional networks inferred from molecular signatures of breast cancer Am J Pathol 2008 172 495 509 10.2353/ajpath.2008.061079 18187569 PMC2312359

[B4] Elkon R Linhart C Sharan R Shamir R Shiloh Y Genome-wide in silico identification of transcriptional regulators controlling the cell cycle in human cells Genome Res 2003 13 773 780 10.1101/gr.947203 12727897 PMC430898

[B5] Pilpel Y Sudarsanam P Church GM Identifying regulatory networks by combinatorial analysis of promoter elements Nat Genet 2001 29 153 159 10.1038/ng724 11547334

[B6] Luscombe NM Babu MM Yu H Snyder M Teichmann SA Gerstein M Genomic analysis of regulatory network dynamics reveals large topological changes Nature 2004 431 308 312 10.1038/nature02782 15372033

[B7] Chen F Castranova V Shi X New insights into the role of nuclear factor-kappaB in cell growth regulation 2001 159 387 397 10.1016/s0002-9440(10)61708-7 PMC1850555 11485895

[B8] Biswas DK Shi Q Baily S Strickland I Ghosh S Pardee AB Iglehart JD NF-kappa B activation in human breast cancer specimens and its role in cell proliferation and apoptosis Proc Natl Acad Sci USA 2004 101 10137 10142 10.1073/pnas.0403621101 15220474 PMC454178

[B9] Sørlie T Perou CM Tibshirani R Aas T Geisler S Johnsen H Hastie T Eisen MB Van De Rijn M Jeffrey SS Thorsen T Quist H Matese JC Brown PO Botstein D Lønning PE Børresen-Dale A-L Gene expression patterns of breast carcinomas distinguish tumor subclasses with clinical implications Proc Natl Acad Sci USA 2001 98 10869 10874 10.1073/pnas.191367098 11553815 PMC58566

[B10] Muggerud AA Johnsen H Barnes DA Steel A Lønning PE Naume B Sørlie T Børresen-Dale A-L Evaluation of MetriGenix custom 4D™arrays applied for detection of breast cancer subtypes BMC Cancer 2006 6 59 10.1186/1471-2407-6-59 16536878 PMC1421426

[B11] Nikitin A Pathway studio–the analysis and navigation of molecular networks Bioinformatics 2003 19 2155 2157 10.1093/bioinformatics/btg290 14594725

[B12] Scherf M Epple A Werner T The next generation of literature analysis: Integration of genomic analysis into text mining Brief Bioinform 2005 6 287 297 10.1093/bib/6.3.287 16212776

[B13] Cartharius K Frech K Grote K Klocke B Haltmeier M Klingenhoff A Frisch M Bayerlein M Werner T MatInspector and beyond: promoter analysis based on transcription factor binding sites Bioinformatics 2005 21 2933 2942 10.1093/bioinformatics/bti473 15860560

[B14] Jolliffe IT Principal Component Analysis Chemom Intell Lab Syst 1986 2 37 52

[B15] Enerly E Steinfeld I Kleivi K Leivonen S-K Aure MR Russnes HG Rønneberg JA Johnsen H Navon R Rødland E Mäkelä R Naume B Perälä M Kallioniemi O Kristensen VN Yakhini Z Børresen-Dale A-L miRNA-mRNA Integrated Analysis Reveals Roles for miRNAs in Primary Breast Tumors PLoS One 2011 6 13 10.1371/journal.pone.0016915 PMC3043070 21364938

[B16] Parker JS Mullins M Cheang MCU Leung S Voduc D Vickery T Davies S Fauron C He X Hu Z Quackenbush JF Stijleman IJ Palazzo J Marron JS Nobel AB Mardis E Nielsen TO Ellis MJ Perou CM Bernard PS Supervised risk predictor of breast cancer based on intrinsic subtypes J Clin Oncol 2009 27 1160 1167 10.1200/JCO.2008.18.1370 19204204 PMC2667820

[B17] Hwang I Choi YS Jeon M-Y Jeong S NF-κB p65 represses β-catenin-activated transcription of cyclin D1 Biochem Biophys Res Commun 2010 403 79 84 10.1016/j.bbrc.2010.10.118 21056029

[B18] Johansson H Svensson F Runnberg R Simonsson T Simonsson S Phosphorylated nucleolin interacts with translationally controlled tumor protein during mitosis and with Oct4 during interphase in ES cells PLoS One 2010 5 e13678 10.1371/journal.pone.0013678 21048921 PMC2965110

[B19] Tsai RYL McKay RDG A nucleolar mechanism controlling cell proliferation in stem cells and cancer cells Genes Dev 2002 16 2991 3003 10.1101/gad.55671 12464630 PMC187487

[B20] Kel-Margoulis OV Romashchenko AG Kolchanov NA Wingender E Kel AE COMPEL: a database on composite regulatory elements providing combinatorial transcriptional regulation Nucleic Acids Res 2000 28 311 315 10.1093/nar/28.1.311 10592258 PMC102399

[B21] Klingenhoff A Frech K Quandt K Werner T Functional promoter modules can be detected by formal models independent of overall nucleotide sequence similarity Bioinformatics 1999 15 180 186 10.1093/bioinformatics/15.3.180 10222404

[B22] Fessele S Maier H Zischek C Nelson PJ Werner T Regulatory context is a crucial part of gene function Trends Genet 2002 18 60 63 10.1016/S0168-9525(02)02591-X 11818130

[B23] Oikawa T Yamada T Molecular biology of the Ets family of transcription factors Gene 2003 303 11 34 12559563 10.1016/s0378-1119(02)01156-3

[B24] Oikawa T ETS transcription factors: possible targets for cancer therapy Cancer Sci 2004 95 626 633 10.1111/j.1349-7006.2004.tb03320.x 15298723 PMC11159856

[B25] Sampath J Sun D Kidd VJ Grenet J Gandhi A Shapiro LH Wang Q Zambetti GP Schuetz JD Mutant p53 cooperates with ETS and selectively up-regulates human MDR1 not MRP1 J Biol Chem 2001 276 39359 39367 10.1074/jbc.M103429200 11483599

[B26] Dittmer J The Biology of the Ets1 Proto-Oncogene Mol Cancer 2003 2 29 10.1186/1476-4598-2-29 12971829 PMC194255

[B27] De Siervi A De Luca P Moiola C Gueron G Tongbai R Chandramouli GVR Haggerty C Dzekunova I Petersen D Kawasaki E Kil WJ Camphausen K Longo D Gardner K Identification of new Rel/NFkappaB regulatory networks by focused genome location analysis Cell cycle Georgetown Tex 2009 8 2093 2100 10.4161/cc.8.13.8926 PMC2809250 19502793

[B28] Gu L Zhu N Findley HW Woods WG Zhou M Identification and characterization of the IKKalpha promoter: positive and negative regulation by ETS-1 and p53, respectively J Biol Chem 2004 279 52141 52149 10.1074/jbc.M407915200 15469934

[B29] Sementchenko VI Watson DK Ets target genes: past, present and future Oncogene 2000 19 6533 6548 10.1038/sj.onc.1204034 11175369

[B30] Ryan KM Ernst MK Rice NR Vousden KH Role of NF-kappaB in p53-mediated programmed cell death Nature 2000 404 892 897 10.1038/35009130 10786798

[B31] Guo G Wang T Gao Q Tamae D Wong P Chen T Chen W-C Shively JE Wong JYC Li JJ Expression of ErbB2 enhances radiation-induced NF-kappaB activation Oncogene 2004 23 535 545 10.1038/sj.onc.1207149 14724581

[B32] Pianetti S Arsura M Romieu-Mourez R Coffey RJ Sonenshein GE Her-2/neu overexpression induces NF-kappaB via a PI3-kinase/Akt pathway involving calpain-mediated degradation of IkappaB-alpha that can be inhibited by the tumor suppressor PTEN Oncogene 2001 20 1287 1299 10.1038/sj.onc.1204257 11313873

[B33] Ray P Ghosh SK Zhang DH Ray A Repression of interleukin-6 gene expression by 17 beta-estradiol: inhibition of the DNA-binding activity of the transcription factors NF-IL6 and NF-kappa B by the estrogen receptor FEBS Lett 1997 409 79 85 10.1016/S0014-5793(97)00487-0 9199508

[B34] Wang CY Cusack JC Liu R Baldwin AS Control of inducible chemoresistance: enhanced anti-tumor therapy through increased apoptosis by inhibition of NF-kappaB Nat Med 1999 5 412 417 10.1038/7410 10202930

[B35] Kantidze OL Kamalyukova IM Razin SV Association of the mammalian transcriptional regulator kaiso with centrosomes and the midbody Cell cycle Georgetown Tex 2009 8 2303 2304 10.4161/cc.8.14.8944 19502788

[B36] Tong G-X Yu WM Beaubier NT Weeden EM Hamele-Bena D Mansukhani MM O’Toole KM Expression of PAX8 in normal and neoplastic renal tissues: an immunohistochemical study Modern pathology an official journal of the United States and Canadian Academy of Pathology Inc 2009 22 1218 1227 10.1038/modpathol.2009.88 19525927

[B37] Liu H Hew HC Lu Z-G Yamaguchi T Miki Y Yoshida K DNA damage signalling recruits RREB-1 to the p53 tumour suppressor promoter Biochem J 2009 422 543 551 10.1042/BJ20090342 19558368

[B38] Ritchie A Gutierrez O Fernandez-Luna JL PAR bZIP-bik is a novel transcriptional pathway that mediates oxidative stress-induced apoptosis in fibroblasts Cell Death Differ 2009 16 838 846 10.1038/cdd.2009.13 19219069

[B39] Frank DA STAT3 as a central mediator of neoplastic cellular transformation Cancer Lett 2007 251 199 210 10.1016/j.canlet.2006.10.017 17129668

[B40] Felty Q Xiong W-C Sun D Sarkar S Singh KP Parkash J Roy D Estrogen-induced mitochondrial reactive oxygen species as signal-transducing messengers Biochemistry 2005 44 6900 6909 10.1021/bi047629p 15865435

[B41] Kunkle B Felty Q Trevino F Roy D Oncomine meta-analysis of breast cancer microarray data identifies upregulation of NRF-1 expression in human breast carcinoma Distribution 2009 715 719

[B42] Ho Sui SJ Mortimer JR Arenillas DJ Brumm J Walsh CJ Kennedy BP Wasserman WW oPOSSUM: identification of over-represented transcription factor binding sites in co-expressed genes Nucleic Acids Res 2005 33 3154 3164 10.1093/nar/gki624 15933209 PMC1142402

[B43] Tharakaraman K Bodenreider O Landsman D Spouge JL Mariño-Ramírez L The biological function of some human transcription factor binding motifs varies with position relative to the transcription start site Nucleic Acids Res 2008 36 2777 2786 10.1093/nar/gkn137 18367472 PMC2377430

