# Supplementary material for: Overrepresentation of transcription factor families in the genesets underlying breast cancer subtypes
Source: BMC Genomics. 2012 May 22;13:199. doi: 10.1186/1471-2164-13-199 (PMC3441847; doi:10.1186/1471-2164-13-199)
Supplement: Additional file 1 — Table S1. Subtype-specific gene list. Table shows the 197 subtype-specific best discriminatory genes, which is a subset of the intrinsic gene-list. [file 1471-2164-13-199-S1.pdf]

Supplementary Table 1: Table shows the 197 subtype-specific best discriminatory genes, which is a subset of the intrinsic gene-list.

Subtype	Name	Symbol
**LUMINAL A**	Acyl-Coenzyme A dehydrogenase, short/branched chain	ACADSB
	Adrenergic, alpha-2A-, receptor	ADRA2A
	Angiotensin II receptor, type 1	AGTR1
	Activated leukocyte cell adhesion molecule	ALCAM
	Annexin A9	ANXA9
	N-acylsphingosine amidohydrolase (acid ceramidase) 1	ASAH1
	Beclin 1, autophagy related	BECN1
	Complement factor B	CFB
	Biliverdin reductase A	BLVRA
	Chromosome 14 open reading frame 132	C14orf132
	Complement component 4B (Childo blood group)	C4A
	Calcium/calmodulin-dependent protein kinase II inhibitor 1	CAMK2N1
	Cyclin D1	CCND1
	Cytochrome c oxidase subunit VIc	COX6C
	Carnitine acetyltransferase	CRAT
	Cytochrome b5 type A (microsomal)	CYB5A
	Cytochrome P450, family 2, subfamily A, polypeptide 6	CYP2A6
	Adaptor protein, phosphotyrosine interaction, PH domain and leucine zipper containing 2	APPL2
	Receptor accessory protein 5	REEP5
	Ectonucleotide pyrophosphatase/phosphodiesterase 5 (putative function)	ENPP5
	Estrogen receptor 1	ESR1
	Fructose-1,6-bisphosphatase 1	FBP1
	Enoyl Coenzyme A hydratase domain containing 2	ECHDC2
	Acyl-Coenzyme A binding domain containing 4	ACBD4
	Fms-related tyrosine kinase 1 (vascular endothelial growth factor/vascular permeability factor receptor)	FLT1
	Flavin containing monooxygenase 5	FMO5
	Fibromodulin	FMOD
	Forkhead box A1	FOXA1
	UDP-N-acetyl-alpha-D-galactosamine:polypeptide N-acetylgalactosaminyltransferase 10 (GalNAc-T10)	GALNT10
	GATA binding protein 3	GATA3
	Glucocorticoid receptor DNA binding factor 1	GRLF1
	Glutathione S-transferase M3 (brain)	GSTM3
	Hexamethylene bis-acetamide inducible 1	HEXIM1
	Hydroxysteroid (17-beta) dehydrogenase 4	HSD17B4
	KIAA0182	KIAA0182
	PHD finger protein 15	PHF15
	Jumonji domain containing 2B	JMJD2B
	Mediator complex subunit 13-like	MED13L
	Solute carrier family 39 (zinc transporter), member 6	SLC39A6
	Nephronectin	NPNT
	LPS-responsive vesicle trafficking, beach and anchor containing	LRBA
	Basal cell adhesion molecule (Lutheran blood group)	BCAM
	Methylcrotonoyl-Coenzyme A carboxylase 2 (beta)	MCCC2
	Chromosome 10 open reading frame 32	C10orf32
	Sushi, nidogen and EGF-like domains 1	SNED1
	Mahogunin, ring finger 1	MGRN1
	Msh homeobox 2	MSX2
	Mucin 1, cell surface associated	MUC1
	N-acetyltransferase 1 (arylamine N-acetyltransferase)	NAT1
	Transcribed locus	IMAGE:132012
	Neuropeptide Y receptor Y1	NPY1R
	Plasminogen activator, tissue	PLAT
	Sushi, von Willebrand factor type A, EGF and pentraxin domain containing 1	SVEP1

Subtype	Name	Symbol
	protein tyrosine phosphatase type IVA, member 2	PTP4A2
	Protein tyrosine phosphatase, receptor type, N polypeptide 2	PTPRN2
	Quinoid dihydropteridine reductase	QDPR
	Rabaptin, RAB GTPase binding effector protein 1	RABEP1
	Ral GEF with PH domain and SH3 binding motif 1	RALGPS1
	Retinoic acid receptor responder (tazarotene induced) 3	RARRES3
	RAS-like, estrogen-regulated, growth inhibitor	RERG
	Transcribed locus, strongly similar to NP_003608.1 regulator of G-protein signalling 5 [Homo sapiens]	BU521978
	Sodium channel, nonvoltage-gated 1 alpha	SCNN1A
	Signal peptide, CUB domain, EGF-like 2	SCUBE2
	Selenium binding protein 1	SELENBP1
	Seven in absentia homolog 2 (Drosophila)	SIAH2
	Solute carrier family 40 (iron-regulated transporter), member 1	SLC40A1
	SHC (Src homology 2 domain containing) transforming protein 2	SHC2
	Six transmembrane epithelial antigen of the prostate 2	STEAP2
	Transcription elongation factor A (SII), 3	TCEA3
	Transcription elongation factor A (SII)-like 1	TCEAL1
	Trefoil factor 3 (intestinal)	TFF3
	Transducin-like enhancer of split 3 (E(sp1) homolog, Drosophila)	TLE3
	Vav 3 guanine nucleotide exchange factor	VAV3
	X-box binding protein 1	XBP1
	MRNA; cDNA DKFZp434E033 (from clone DKFZp434E033)	AL080130
	Sorting nexin 13	SNX13
	Endothelin converting enzyme 1	ECE1
	Chromosome 4 open reading frame 18	C4orf18
**LUMINAL B**	Glycine dehydrogenase (decarboxylating)	GLDC
	Syndecan 2	SDC2
	Glutathione S-transferase pi	GSTP1
	Minichromosome maintenance complex component 3	MCM3
	Procollagen-lysine 1, 2-oxoglutarate 5-dioxygenase 1	PLOD1
	Serpin peptidase inhibitor, clade H (heat shock protein 47), member 1, (collagen binding protein 1)	SERPINH1
	PTK7 protein tyrosine kinase 7	PTK7
	Neuregulin 1	NRG1
	Prolyl endopeptidase	PREP
	Replication factor C (activator 1) 3, 38kDa	RFC3
	NIMA (never in mitosis gene a)-related kinase 4	NEK4
	Ribosomal protein L10	RPL10
	THAP domain containing 11	THAP11
	Succinate dehydrogenase complex, subunit A, flavoprotein (Fp)	SDHA
	Transmembrane protein 65	TMEM65
	Chromosome 18 open reading frame 21	C18orf21
	CDGSH iron sulfur domain 1	CISD1
	CLP1, cleavage and polyadenylation factor I subunit, homolog (S. cerevisiae)	CLP1
	ATP-binding cassette, sub-family D (ALD), member 3	ABCD3
	Adenylosuccinate lyase	ADSL
	BTG family, member 3	BTG3
	ATP synthase, H+ transporting, mitochondrial F0 complex, subunit C1 (subunit 9)	ATP5G1
	Cell division cycle 123 homolog (S. cerevisiae)	CDC123
	Prion protein interacting protein	PRNPIP
	EBNA1 binding protein 2	EBNA1BP2
	Transcribed locus, strongly similar to NP_113751.3 Y box protein 1 [Rattus norvegicus]	BM555523
	Gamma-glutamyl hydrolase (conjugase, folylpolygammaglutamyl hydrolase)	GGH
	Lysosomal associated protein transmembrane 4 beta	LAPTM4B
	Peroxiredoxin 4	PRDX4
	Cornichon homolog 4 (Drosophila)	CNIH4

Subtype	Name	Symbol
	Glycyl-tRNA synthetase	GARS
	5'-nucleotidase domain containing 2	NT5DC2
	Thymosin beta 10	TMSB10
	KDEL (Lys-Asp-Glu-Leu) endoplasmic reticulum protein retention receptor 2	KDELR2
	Tweety homolog 3 (Drosophila)	TTYH3
	Nuclear receptor binding factor 2	NRBF2
	Transcribed locus	AL576538
	Acidic (leucine-rich) nuclear phosphoprotein 32 family, member E	ANP32E
	Polymerase (RNA) II (DNA directed) polypeptide F	POLR2F
	Squalene epoxidase	SQLE
	S100 calcium binding protein A10	S100A10
	ASF1 anti-silencing function 1 homolog A (S. cerevisiae)	ASF1A
	Ladinin 1	LAD1
	Serine/threonine kinase 24 (STE20 homolog, yeast)	STK24
	V-maf musculoaponeurotic fibrosarcoma oncogene homolog G (avian)	MAFG
	CTP synthase	CTPS
	Thymosin beta15b	MGC39900
	Cullin 1	CUL1
	Tumor protein p53 binding protein, 2	TP53BP2
	Carbonyl reductase 1	CBR1
	ADAM metallopeptidase domain 9 (meltrin gamma)	ADAM9
	Transcribed locus	BU167807
	Protein tyrosine phosphatase, receptor type, K	PTPRK
	S100 calcium binding protein A11	S100A11
	Calcium and integrin binding family member 2	CIB2
**NORMAL-LIKE**	Rap guanine nucleotide exchange factor (GEF) 3	RAPGEF3
	Acyl-CoA synthetase short-chain family member 2	ACSS2
	RAB11 family interacting protein 5 (class I)	RAB11FIP5
	Trophoblast-derived noncoding RNA	TncRNA
	Phosphoinositide-3-kinase, regulatory subunit 1 (alpha)	PIK3R1
	Aldo-keto reductase family 1, member C1 (dihydrodiol dehydrogenase 1; 20-alpha (3-alpha)-hydroxysteroid dehydrogenase)	AKR1C1
	Acyl-CoA synthetase long-chain family member 1	ACSL1
	Peptidylglycine alpha-amidating monooxygenase	PAM
	Hypothetical LOC653354	LOC653354
	Guanine nucleotide binding protein (G protein), beta polypeptide 2-like 1	GNB2L1
	HCG18290	hCG_18290
	Invasion inhibitory protein 45	RP5-1077B9.4
	Transcribed locus	BG696892
	Protein tyrosine phosphatase, receptor type, M	PTPRM
	Lactotransferrin	LTF
	Aquaporin 3 (Gill blood group)	AQP3
	Actin binding LIM protein 1	ABLIM1
	Transcribed locus	AI546919
	Glutathione S-transferase A4	GSTA4
		IMAGE:161998
	Keratin 13	KRT13
	Transcription factor AP-2 gamma (activating enhancer binding protein 2 gamma)	TFAP2C
	Carbonic anhydrase II	CA2
**BASAL**	Exostoses (multiple) 2	EXT2
	Zinc finger protein 532	ZNF532
	Cyclin-dependent kinase 6	CDK6
	CDNA clone IMAGE:3683736	BX647680
	Chemokine (C-X-C motif) ligand 1 (melanoma growth stimulating activity, alpha)	CXCL1
	Cadherin 3, type 1, P-cadherin (placental)	CDH3
	Secretory leukocyte peptidase inhibitor	SLPI
	Vestigial like 1 (Drosophila)	VGLL1

Subtype	Name	Symbol
	Gamma-aminobutyric acid (GABA) A receptor, pi	GABRP
	Annexin A8-like 1	ANXA8L1
	Keratin 5 (epidermolysis bullosa simplex, Dowling-Meara/Kobner/Weber-Cockayne types)	KRT5
	Tripartite motif-containing 29	TRIM29
	Keratin 17	KRT17
	Milk fat globule-EGF factor 8 protein	MFGE8
	Milk fat globule-EGF factor 8 protein	MFGE8
	Chemokine (C-X3-C motif) ligand 1	CX3CL1
	Frizzled homolog 7 (Drosophila)	FZD7
	Forkhead box C1	FOXC1
	Chitinase 3-like 2	CHI3L2
	Desmocollin 2	DSC2
	Chromosome 1 open reading frame 198	C1orf198
	UDP-GlcNAc:betaGal beta-1,3-N-acetylglucosaminyltransferase 5	B3GNT5
	Solute carrier family 5 (sodium-dependent vitamin transporter), member 6	SLC5A6
	3-ketodihydrosphingosine reductase	KDSR
	Nucleolin	NCL
	Actin, gamma 2, smooth muscle, enteric	ACTG2
	Vinculin	VCL
	Sarcoglycan, epsilon	SGCE
**ERBB2+**	Chromosome 17 open reading frame 63	C17orf63
	Flotillin 2	FLOT2
	SWI/SNF related, matrix associated, actin dependent regulator of chromatin, subfamily e, member 1	SMARCE1
	Tousled-like kinase 1	TLK1
	Mediator complex subunit 24	MED24
	Transcribed locus	BM455010
	V-erb-b2 erythroblastic leukemia viral oncogene homolog 2, neuro/glioblastoma derived oncogene homolog (avian)	ERBB2
	Growth factor receptor-bound protein 7	GRB7
	StAR-related lipid transfer (START) domain containing 3	STARD3
	TBP-like 1	TBPL1
	Carcinoembryonic antigen-related cell adhesion molecule 6 (non-specific cross reacting antigen)	CEACAM6
	S100 calcium binding protein P	S100P
	Hypothetical protein LOC90110	LOC90110
