# Supplementary figures and images for: Overrepresentation of transcription factor families in the genesets underlying breast cancer subtypes
Source: BMC Genomics. 2012 May 22;13:199. doi: 10.1186/1471-2164-13-199 (PMC3441847; doi:10.1186/1471-2164-13-199)

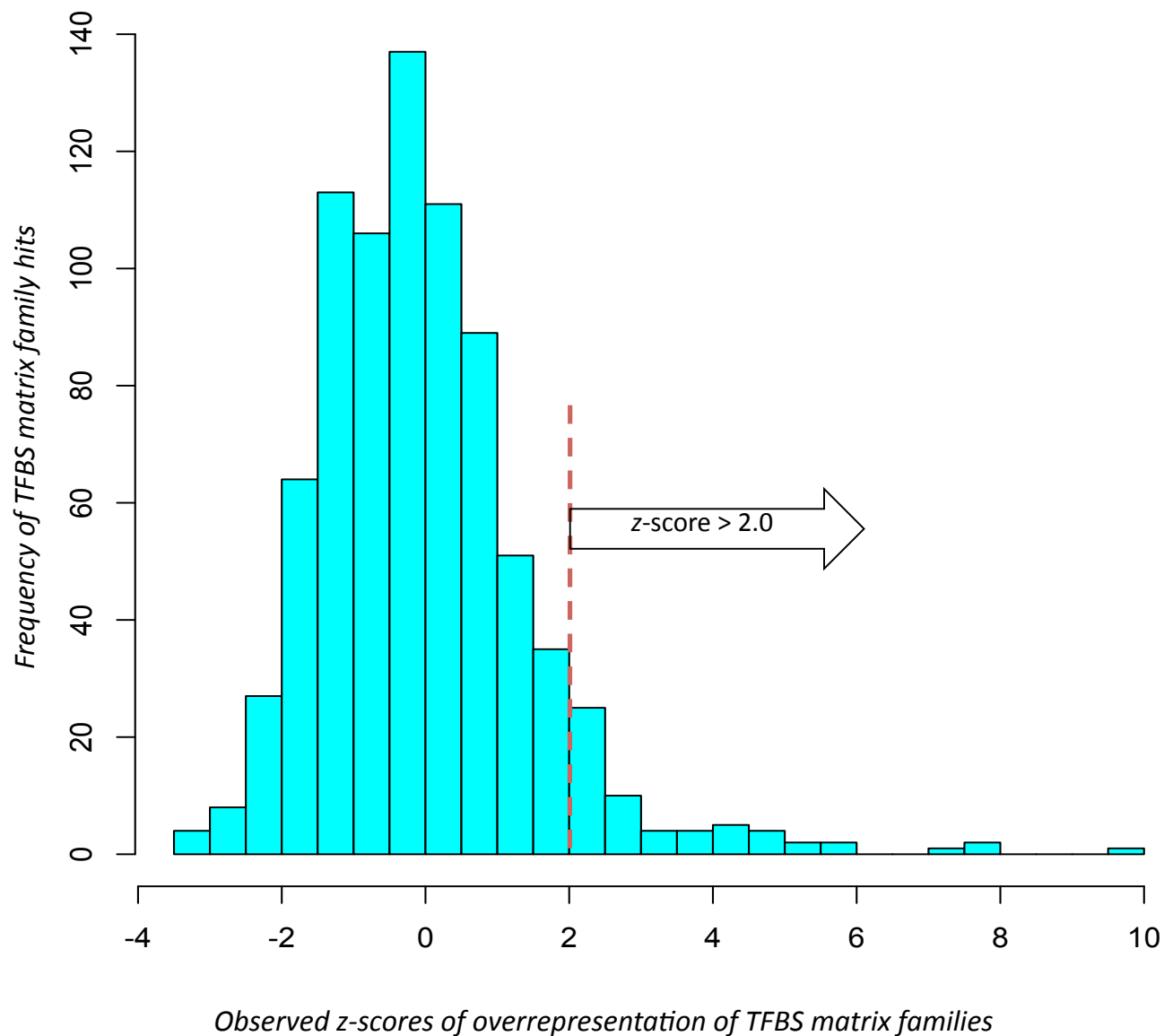

Supplement: Additional file 2 — Figure S1. Histogram of z-scores of overrepresentation. Histogram of TFBS matrix family overrepresentation observed in subtype-specific promoters compared to the reference genomic promoter background shown as z-scores. [file 1471-2164-13-199-S2.pdf]

## Luminal A

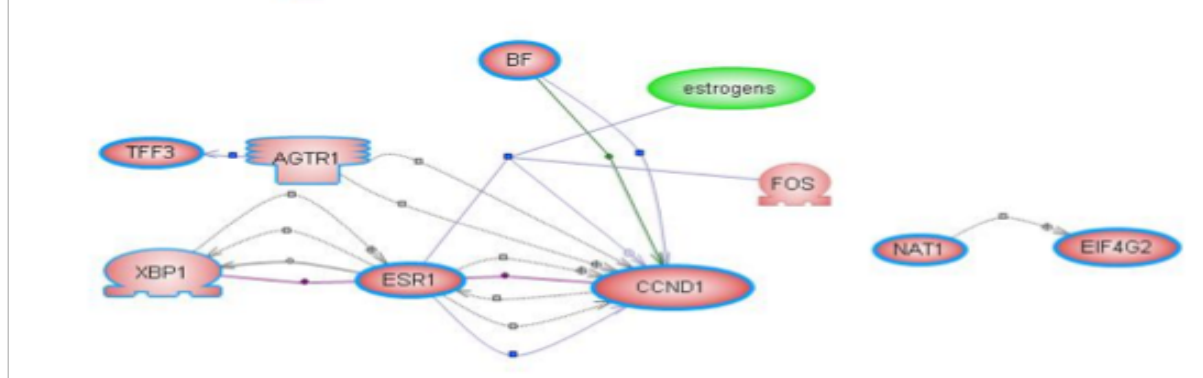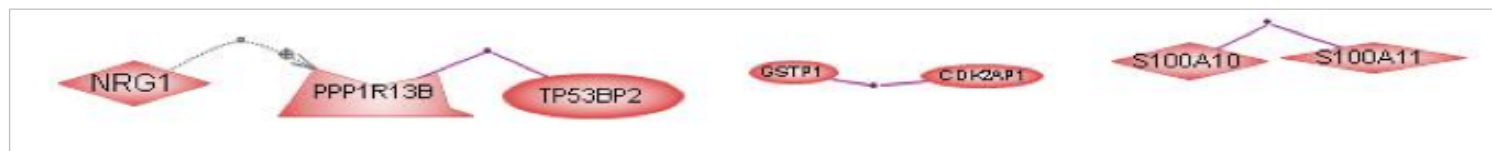

## Luminal B

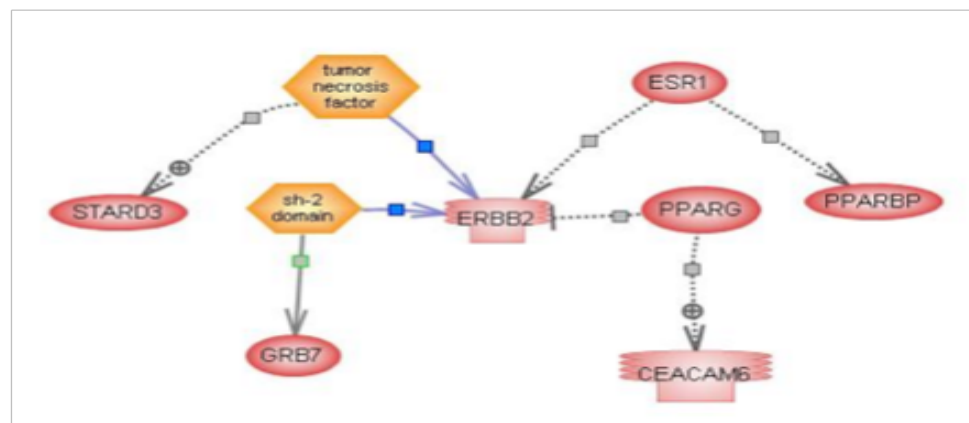

## ERBB2

Supplement: Additional file 3 — Figure S2. Direct interactions between genes defining subtypes. Subtype-relevant key driver interactions for Luminal A, B and ERBB2+ subtypes. [file 1471-2164-13-199-S3.pdf]

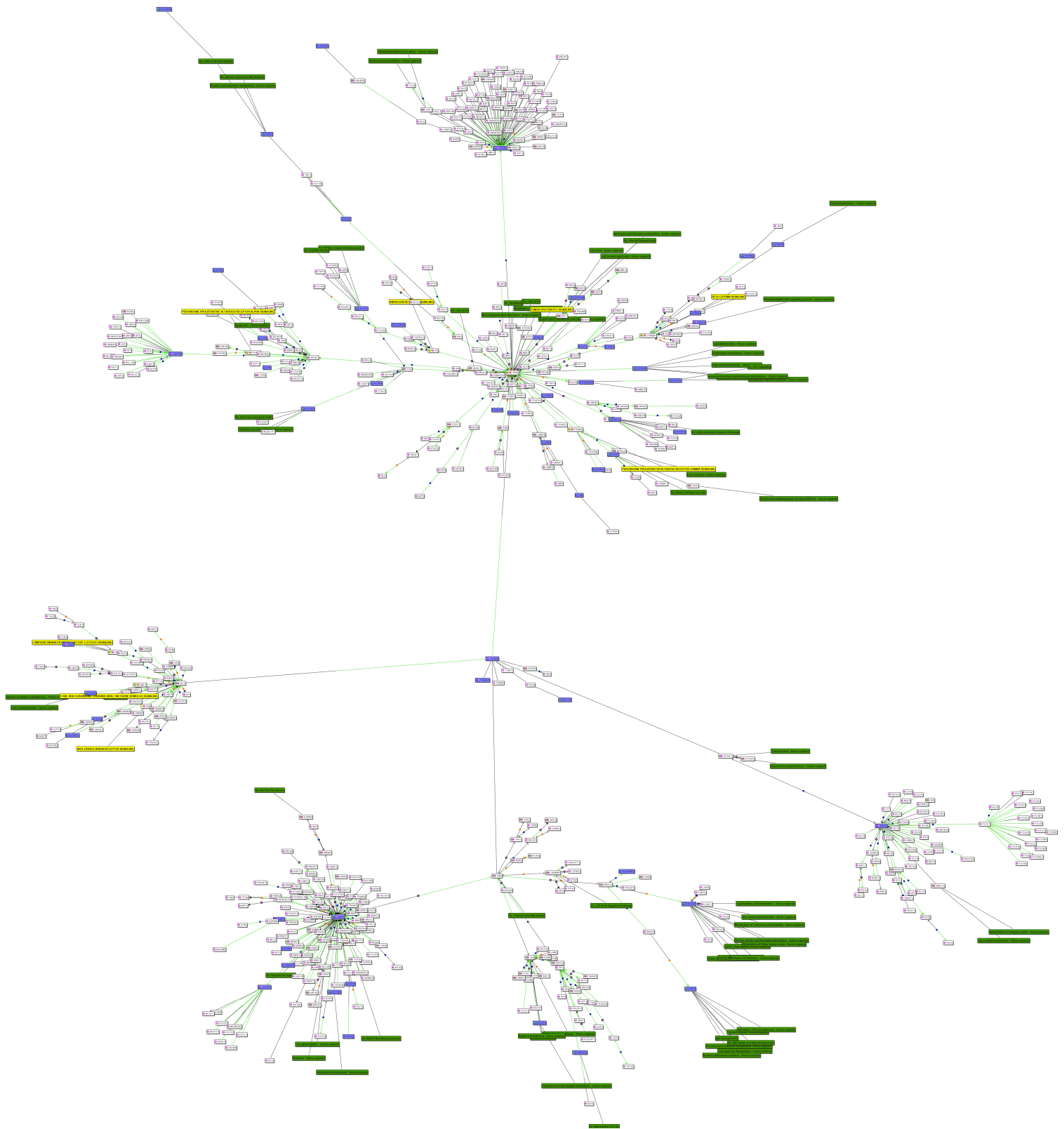

Supplement: Additional file 4 — Figure S3. Protein-protein interactions and TF interactions associated with Luminal A subtype. Network shown here is based on the luminal A specific genelist. [file 1471-2164-13-199-S4.pdf]

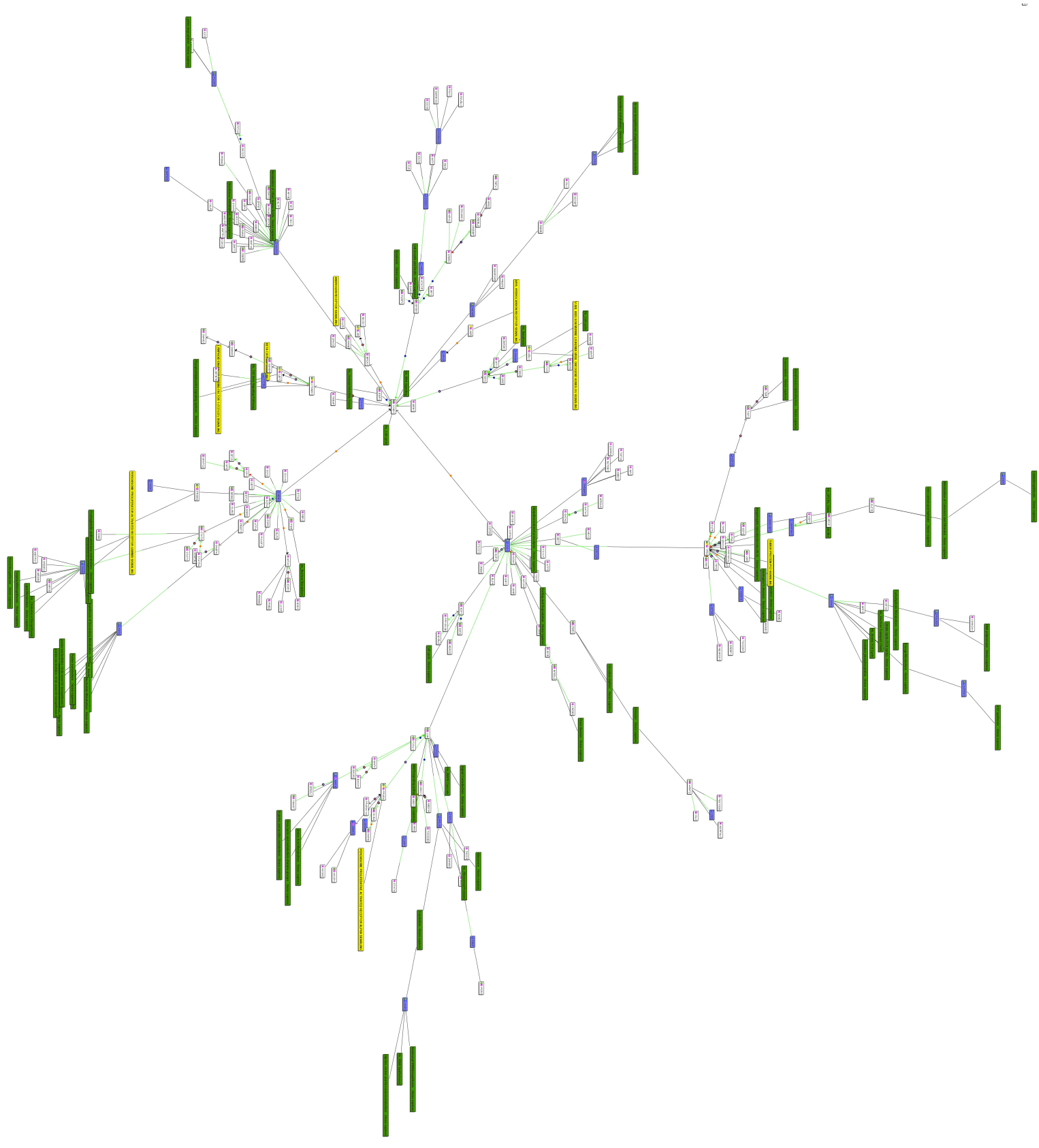

Supplement: Additional file 5 — Figure S4. Protein-protein interactions and TF interactions associated with Luminal B subtype. Network shown here is based on the luminal B specific genelist. [file 1471-2164-13-199-S5.pdf]

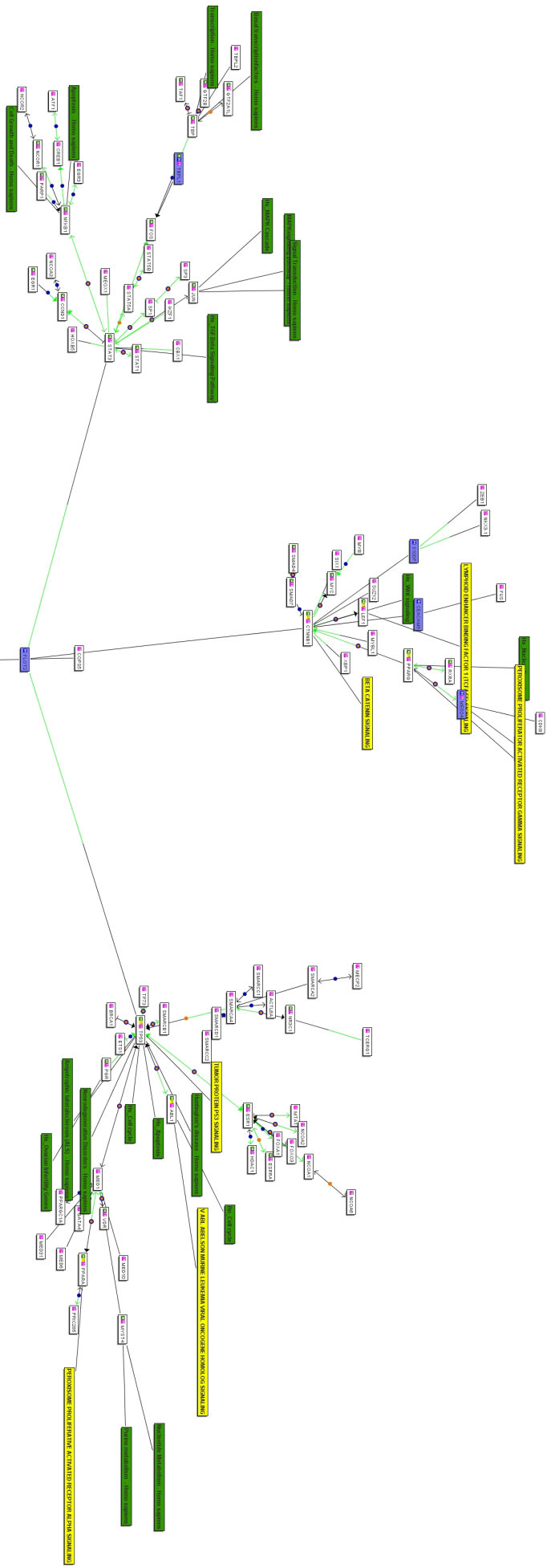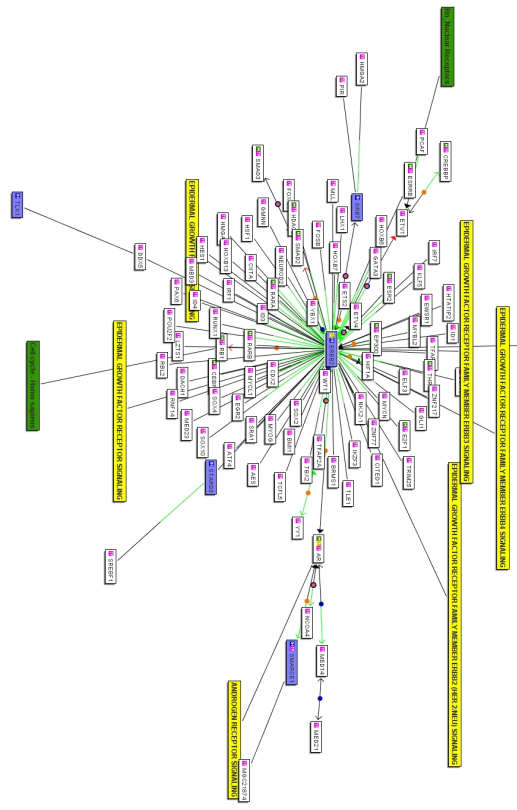

Supplement: Additional file 6 — Figure S5. Protein-protein interactions and TF interactions associated with ERBB2+ subtype. Network shown here is based on the ERBB2+ subtype-specific genelist. [file 1471-2164-13-199-S6.pdf]

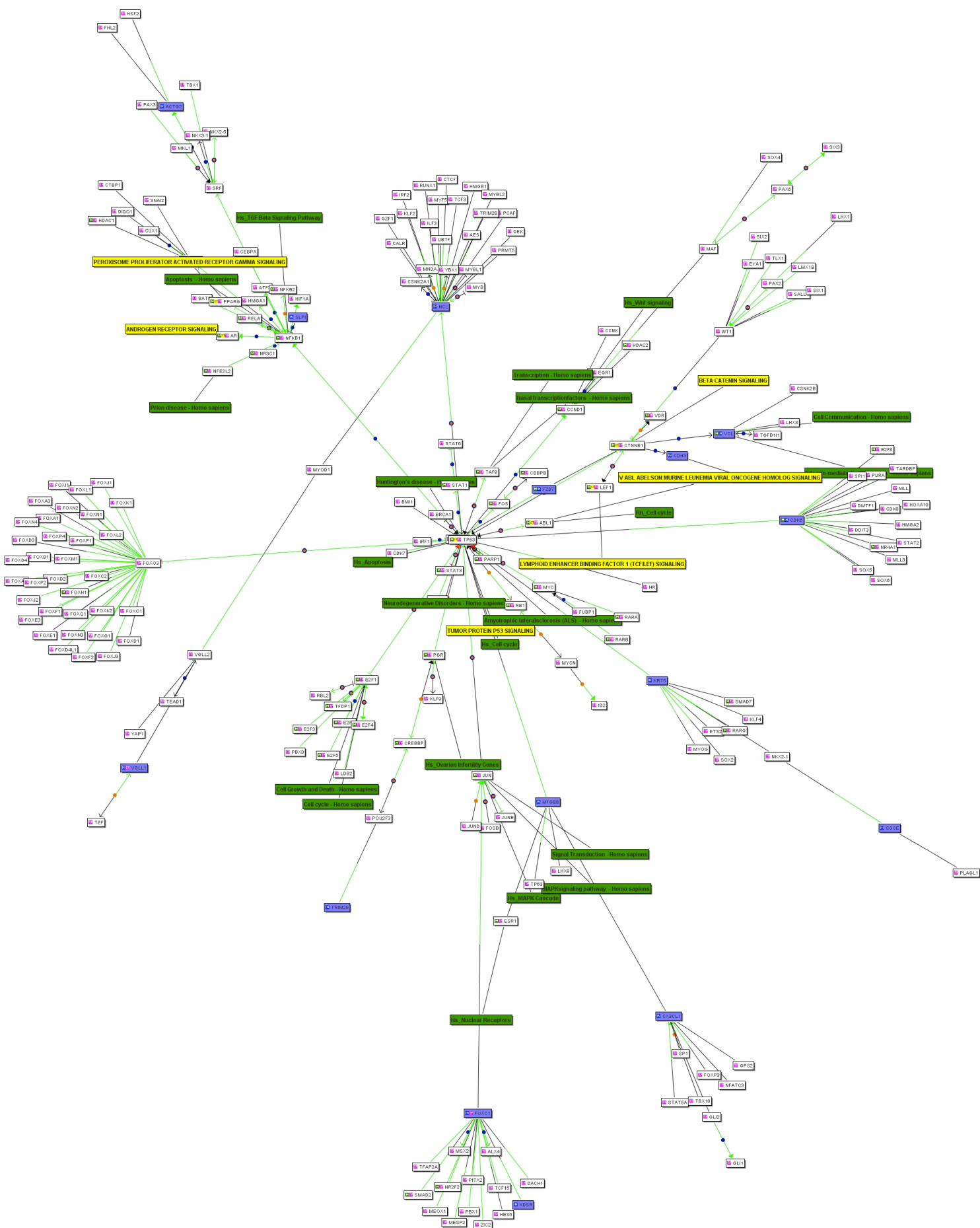

Supplement: Additional file 7 — Figure S6. Protein-protein interactions and TF interactions associated with basal subtype. Network shown here is based on the basal subtype-specific genelist. [file 1471-2164-13-199-S7.pdf]

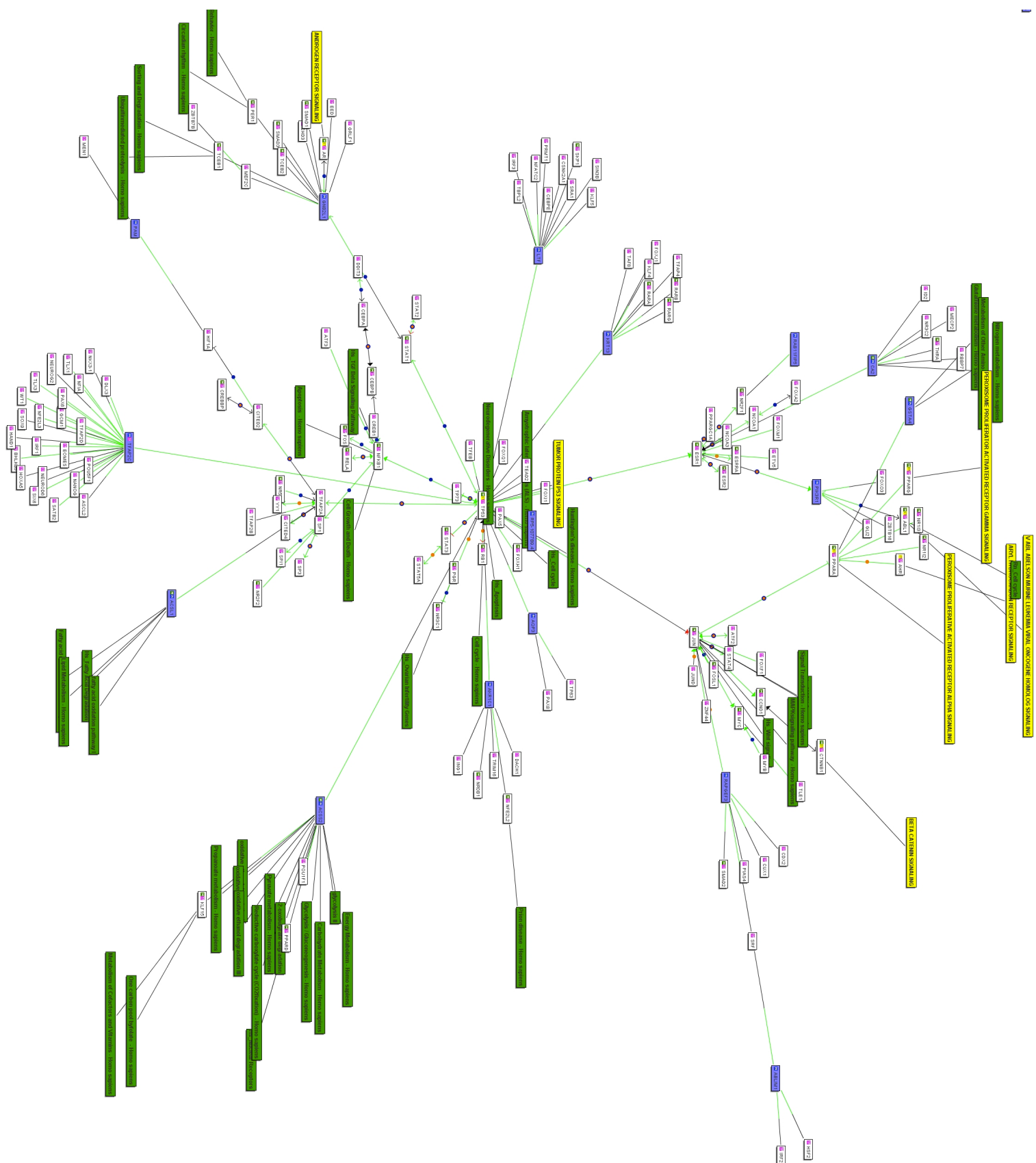

Supplement: Additional file 8 — Figure S7. Protein-protein interactions and TF interactions associated with normal-like subtype. Network shown here is based on the normal-like subtype-specific genelist. [file 1471-2164-13-199-S8.pdf]
