# Supplementary material for: Overrepresentation of transcription factor families in the genesets underlying breast cancer subtypes
Source: BMC Genomics. 2012 May 22;13:199. doi: 10.1186/1471-2164-13-199 (PMC3441847; doi:10.1186/1471-2164-13-199)
Supplement: Additional file 9 — Table S2. TFBS overrepresentation in subtypes-specific gene promoters. List of significantly over-represented transcription factor binding site families in subtypes of breast cancers at the cut-off level of z- score > =2.0. [file 1471-2164-13-199-S9.pdf]

Supplementary Table 2. List of significantly over-represented transcription factor binding site families in subtypes of breast cancers at the cut-off level of z-score >=2.0.

TF Families	Description	Nr of Sequences	Nr of Matches	Expected frequency (genome)	Overrepresentation (genome)	Z-Score (genome)	Expected (promoters)	Overrepresentation (promoters)	Z-Score (promoters)
**Basal**									
O$TF2B	RNA polymerase II transcription factor II B	6	12	0.82	14.71	11.83	6.35	1.89	2.04
V$BTBF	BTB/POZ (broad complex, TramTrack, Bric-a-brac/pox viruses and zinc fingers) transcription factor	12	14	4.51	3.1	4.23	4.38	3.2	4.36
V$MOKF	Mouse Krueppel like factor	14	27	13.68	1.97	3.47	17.43	1.55	2.17
V$SRFF	Serum response element binding factor	17	35	30.62	1.14	0.7	23.52	1.49	2.27
ERBB2+									
V$EKLF	Basic and erythroid krueppel like factors	10	30	6.85	4.38	8.66	17.35	1.73	2.92
V$GREF	Glucocorticoid responsive and related elements	10	24	13.43	1.79	2.75	12.03	2	3.31
V$MAZF	Myc associated zinc fingers	11	32	4.55	7.03	12.64	16.12	1.98	3.83
V$MZF1	Myeloid zinc finger 1 factors	12	24	7.64	3.14	5.74	13.16	1.82	2.85
V$NFKB	Nuclear factor kappa B/c-rel	11	24	7.29	3.29	6	11.67	2.06	3.46
V$PLAG	Pleomorphic adenoma gene	8	16	2.37	6.76	8.54	8.5	1.88	2.4
V$RREB	Ras-responsive element binding protein	10	13	3.62	3.59	4.67	6.83	1.9	2.17
V$SP1F	GC-Box factors SP1/GC	11	58	9.03	6.43	16.15	28.47	2.04	5.45
V$XBBF	X-box binding factors	8	17	7.25	2.35	3.44	10.02	1.7	2.05
V$ZBPF	Zinc binding protein factors	11	63	8.96	7.03	17.9	32.93	1.91	5.16
**Luminal A**									
O$INRE	Core promoter initiator elements	39	54	53.92	1	-0.06	40.17	1.34	2.1
O$MTEN	Core promoter motif ten elements	33	75	9.52	7.88	21.07	57.01	1.32	2.32
O$PTBP	Plant TATA binding protein factor	22	110	114.22	0.96	-0.44	54.75	2.01	7.4
O$XCPE	Activator-, mediator- and TBP-dependent core promoter element for RNA polymerase	39	72	9.72	7.4	19.81	55.89	1.29	2.09
V$AP1F	AP1, Activating protein 1	33	58	55.74	1.04	0.24	43.98	1.32	2.04
V$ARID	AT rich interactive domain factor	10	13	6.94	1.87	2.11	4.56	2.85	3.71
V$CLOX	CLOX and CLOX homology (CDP) factors	69	205	284.23	0.72	-4.74	175.47	1.17	2.19
V$EBOX	E-box binding factors	61	123	56.47	2.18	8.79	98.34	1.25	2.44
V$GATA	GATA binding factors	68	168	205.78	0.82	-2.67	135.12	1.24	2.79
V$GLIF	GLI zinc finger family	54	91	37.79	2.41	8.58	71.41	1.27	2.26
V$HAML	Human acute myelogenous leukemia factors	41	51	39.04	1.31	1.83	36.88	1.38	2.24
V$HNF1	Hepatic Nuclear Factor 1	45	94	119.4	0.79	-2.37	71.19	1.32	2.65
V$HOXF	Factors with moderate activity to homeo domain consensus sequence	80	348	482.2	0.72	-6.16	301.07	1.16	2.68

TF Families	Description	Nr of Sequences	Nr of Matches	Expected frequency (genome)	Overrepresentation (genome)	Z-Score (genome)	Expected (promoters)	Overrepresentation (promoters)	Z-Score (promoters)
V$IRFF	Interferon regulatory factors	62	125	135.18	0.92	-0.92	100.89	1.24	2.35
V$LHXF	Lim homeodomain factors	47	116	172.53	0.67	-4.35	92.05	1.26	2.45
V$MAZF	Myc associated zinc fingers	52	135	31.5	4.29	18.36	111.63	1.21	2.17
V$MEF3	MEF3 binding sites	15	17	8.53	1.99	2.73	10.12	1.68	2
V$MYT1	MYT1 C2HC zinc finger protein	67	157	178.33	0.88	-1.64	125.66	1.25	2.75
V$OAZF	Olfactory associated zinc finger protein	28	54	13.73	3.93	10.73	28.11	1.92	4.79
V$P53F	p53 tumor suppressor	44	89	64.02	1.39	3.06	71.54	1.24	2.01
V$PARF	PAR/bZIP family	62	182	233.26	0.78	-3.4	148.77	1.22	2.69
V$PAX9	PAX-9 binding sites	21	28	5.66	4.95	9.18	17.12	1.64	2.51
V$PLZF	C2H2 zinc finger protein PLZF	34	48	42.96	1.12	0.69	30.25	1.59	3.14
V$SF1F	Vertebrate steroidogenic factor	39	45	31.8	1.42	2.25	32.82	1.37	2.04
V$SP1F	GC-Box factors SP1/GC	62	230	62.48	3.68	21.14	197.13	1.17	2.31
V$ZBPF	Zinc binding protein factors	58	273	62.02	4.4	26.74	228	1.2	2.95
V$ZF5F	ZF5 POZ domain zinc finger	26	83	7.24	11.47	27.98	57.06	1.45	3.37
Luminal B									
O$MTEN	Core promoter motif ten elements	26	63	5.82	10.83	23.51	34.84	1.81	4.69
O$TF2B	RNA polymerase II transcription factor II B	18	28	1.6	17.47	20.46	12.47	2.24	4.26
O$XCPE	Activator-, mediator- and TBP-dependent core promoter element for RNA polymerase II from TATA-less promoters	26	58	5.94	9.76	21.15	34.15	1.7	4
V$AHRR	AHR-arnt heterodimers and AHR-related factors	30	65	16.83	3.86	11.62	38.04	1.71	4.29
V$CDEF	Cell cycle regulators: Cell cycle dependent element	20	32	2.42	13.22	18.69	16.37	1.95	3.74
V$CTCF	CTCF and BORIS gene family, transcriptional regulators with 11 highly conserved zinc finger domains	26	71	5.08	13.98	29.03	40.98	1.73	4.61
V$E2FF	E2F-myc activator/cell cycle regulator	44	159	43.69	3.64	17.38	85.2	1.87	7.95
V$EGRF	EGR/nerve growth factor induced protein C & related factors	37	224	35.37	6.33	31.65	133.2	1.68	7.84
V$EKLF	Basic and erythroid krueppel like factors	37	96	28.98	3.31	12.36	73.42	1.31	2.58
V$MAZF	Myc associated zinc fingers	35	107	19.25	5.56	19.89	68.22	1.57	4.64
V$NRF1	Nuclear respiratory factor 1	23	121	3.24	37.33	65.13	51.11	2.37	9.71
V$PAX1	PAX-1 binding sites	11	11	4.75	2.32	2.64	5.24	2.1	2.3
V$PAX9	PAX-9 binding sites	14	19	3.46	5.5	8.09	10.46	1.82	2.48
V$PLAG	Pleomorphic adenoma gene	29	61	10.02	6.09	15.95	35.98	1.7	4.09
V$SP1F	GC-Box factors SP1/GC	37	166	38.18	4.35	20.62	120.47	1.38	4.11
V$WHNF	Winged helix binding sites	21	30	4.65	6.45	11.52	18.88	1.59	2.44
V$XBBF	X-box binding factors	32	58	30.67	1.89	4.85	42.4	1.37	2.32

TF Families	Description	Nr of Sequences	Nr of Matches	Expected frequency (genome)	Overrepresentation (genome)	Z-Score (genome)	Expected (promoters)	Overrepresentation (promoters)	Z-Score (promoters)
V$ZBPF	Zinc binding protein factors	37	207	37.9	5.46	27.4	139.33	1.49	5.7
V$ZF5F	ZF5 POZ domain zinc finger	25	70	4.42	15.83	30.95	34.87	2.01	5.87
**Normal-like**									
V$NRF1	Nuclear respiratory factor 1	6	28	1.18	23.76	24.25	18.58	1.51	2.07
