# Supplementary material for: Overrepresentation of transcription factor families in the genesets underlying breast cancer subtypes
Source: BMC Genomics. 2012 May 22;13:199. doi: 10.1186/1471-2164-13-199 (PMC3441847; doi:10.1186/1471-2164-13-199)
Supplement: Additional file 10 — Table S3. Over-representation of potential TFBS in subtype-specific promoter sequences. Table shows the fold over-representation of potential transcriptional factor hits (represented as TFBS families) in subtype- specific gene promoter sequences. [file 1471-2164-13-199-S10.pdf]

Supplementary Table 3: Table shows the fold over-representation of potential transcriptional factor hits (represented as TFBS families) in subtype-specific gene promoter sequences.

TFBSFamilyName	Basal	ERBB2+	LumA	LumB	Normal-like
O$INRE	1.04	0.69	1.34	0.65	1.23
O$MTEN	1.47	0.85	1.32	1.81	0.87
O$PTBP	0.94	0.51	2.01	0.6	1.07
O$TF2B	1.89	0.34	0.98	2.24	1.32
O$TF2D	0.81	1.32	0.95	1.35	0.86
O$TF3C	0.67	0	0.83	1.36	0
O$VTBP	1.24	0.75	1.03	0.98	1.28
O$XCPE	0.86	1.61	1.29	1.7	1.05
V$AARF	0.84	0.6	0.95	0.99	1.17
V$AHRR	0.72	0.78	0.85	1.71	0.87
V$AIRE	0.75	0.81	1.11	1.05	1.05
V$AP1F	1.24	0.47	1.32	0.52	1.33
V$AP1R	0.93	0.71	0.97	0.75	1.07
V$AP2F	0.87	1.19	1.3	1.21	1.55
V$AP4R	0.6	0.64	1.06	1.12	0.42
V$ARID	0.7	1.52	2.85	0.36	0
V$ATBF	0.66	0.32	1.01	0.79	0.52
V$BARB	0.91	0.49	1.23	0.92	0.95
V$BCL6	0.82	0.95	1.12	1	1.24
V$BNCF	1.08	1.16	0.67	0.46	1.51
V$BPTF	1.24	1.14	0.66	0.54	0.74
V$BRAC	1.39	0.54	1.17	1.21	1.8
V$BRNF	0.86	0	1.08	0.99	0.96
V$BTBF	3.2	1.97	0.64	0.35	0.96
V$CAAT	0.82	0.73	1.18	1.26	1.1
V$CABL	1.02	0.73	0.63	0.87	0.48
V$CART	0.56	0.45	1.08	1.06	0.58
V$CDEF	0.72	0.78	0.75	1.95	0.67
V$CDXF	1.03	0.69	1.1	1.38	1.62
V$CEBP	1.23	0.63	1.07	1.16	0.82
V$CHOP	1.08	1.75	0.59	1.1	0
V$CHRE	0.8	0.69	0.7	1.31	1.12
V$CHRF	0.55	0.98	0.74	0.6	1.66
V$CIZF	0.84	0.9	1.07	0.85	1.32
V$CLOX	1.1	0.99	1.17	1.03	0.77
V$CP2F	1.51	1.37	1.01	0.85	0.67
V$CREB	0.75	1.02	0.98	0.93	0.98
V$CSEN	0.75	2.01	1.34	0.48	1.31
V$CTCF	1.05	1.34	1.15	1.73	1.07
V$DEAF	1.08	1.54	0.82	1.1	0.5
V$DICE	1.07	0.58	1.12	0.95	0.19
V$DMRT	0.86	0.77	1.13	0.99	0.58
V$DMTF	1.54	0.74	1.17	1.04	0.24
V$E2FF	0.88	0.6	0.97	1.87	0.71
V$E4FF	0.64	1.11	1	1.24	1.44
V$EBOX	0.69	0.84	1.25	1.11	0.69
V$EGRF	0.96	1.24	1.06	1.68	0.85
V$EKLF	1.12	1.73	0.99	1.31	0.75
V$EREF	0.87	1.53	0.96	1.01	1.11
V$ETSF	0.86	1.19	0.92	1.14	0.91
V$EVI1	0.71	0.75	0.92	1.09	1.18
V$FAST	0.82	1.23	0.77	0.74	0.97
V$FKHD	0.98	0.61	1.05	0.79	0.82
V$FXRE	0.7	0	1.04	0.81	1.97
V$GABF	0.16	1.59	1.17	0.92	0.11
V$GATA	1.09	0.72	1.24	0.91	0.9
V$GCMF	1.51	1.08	0.6	0.77	0.47
V$GCNR	0.69	0	0.43	0	0
V$GFI1	0.94	1.18	0.97	0.95	0.55
V$GKLF	0.84	1.47	0.88	1.02	0.88
V$GLIF	1.04	1.07	1.27	1.24	0.57
V$GREF	1.04	2	0.78	0.71	0.86
V$GRHL	0.77	0.71	0.65	0.68	0.93
V$GUCE	1.3	0	1.21	1.32	0
V$GZF1	0.64	0.69	1.34	0.57	0.67
V$HAML	1.22	1.5	1.38	1.06	0.73
V$HAND	0.85	1.1	1.08	0.94	0.79
V$HEAT	1.14	1.22	0.96	0.93	0.75
V$HESF	0.73	0.55	1.08	1.27	0.96
V$HICF	0.54	0.58	1.42	1.36	1.12
V$HIFF	0.55	0.88	0.98	1.11	1.24
V$HMTB	1.62	1.28	1.06	0.95	1.67
V$HNF1	0.68	0.39	1.32	0.8	1.07
V$HNF6	0.55	0.6	1.17	0.93	1.08
V$HOMF	0.76	0.73	1	0.84	1.01
V$HOXC	0.74	0.79	0.88	1.07	0.82
V$HOXF	0.84	0.92	1.16	0.9	0.87
V$HOXH	0.69	0.4	1.09	0.96	1.14
V$IKRS	1.09	0.65	1.13	1.05	0.76
V$INSM	1.31	1.56	0.88	1.03	1.52
V$IRFF	1.05	0.82	1.24	1.25	1.12

TFBSFamilyName	Basal	ERBB2+	LumA	LumB	Normal-like
V$LEFF	0.92	0.93	0.86	1.03	1.29
V$LHXF	0.59	0.53	1.26	0.71	0.78
V$MAZF	0.98	1.98	1.21	1.57	0.93
V$MEF2	1.12	0.71	1.16	0.95	0.99
V$MEF3	1.9	1.37	1.68	1.45	0.44
V$MITF	0.51	1.09	0.89	0.94	0.71
V$MOKF	1.55	1.73	1.27	0.79	1.12
V$MTF1	0.61	0.88	0.83	1.46	0.57
V$MYBL	1.17	0.98	0.93	0.96	1.23
V$MYOD	0.7	1.16	0.77	0.78	0.46
V$MYT1	1.02	0.88	1.25	0.98	0.72
V$MZF1	1.09	1.82	0.9	1.08	1.04
V$NBRE	0.81	1.39	1.21	0.58	1.13
V$NEUR	0.47	0.76	0.98	0.68	1.31
V$NF1F	1	0.94	1.01	0.83	0.88
V$NFAT	0.59	0.68	1.1	1.04	0.76
V$NFKB	0.76	2.06	1.1	0.95	1.23
V$NKX6	0.82	0.65	1.19	0.55	0.97
V$NKXH	1.02	0.91	1.06	0.99	0.98
V$NOLF	1.25	0.85	1.18	0.63	0.46
V$NR2F	1.02	0.75	1.1	0.79	0.83
V$NRF1	0.81	0.58	0.97	2.37	1.51
V$NRSF	0.52	0.48	0.96	1.3	0.42
V$OAZF	1.49	1.72	1.92	1.05	0.96
V$OCT1	1.05	0.45	0.91	0.74	1.15
V$OCTB	0.6	0	1.22	0.43	1.17
V$P53F	1.08	0.39	1.24	0.94	0.94
V$PARF	0.89	0.98	1.22	1.06	1.15
V$PAX1	1.5	2.42	1.63	2.1	0.53
V$PAX2	1.45	0.7	1.31	0.74	0.91
V$PAX3	1.22	1.2	1.14	1.19	1.09
V$PAX4	0	0	0	0	3.97
V$PAX5	0.86	0.52	1.19	1.14	1.07
V$PAX6	0.84	1.18	1.15	1.15	1.13
V$PAX8	1.48	2.13	0.73	0.75	1.38
V$PAX9	1.5	0.81	1.64	1.82	0.79
V$PBXC	1.15	1.02	0.76	0.82	1.32
V$PDX1	0.7	0.7	1.1	0.97	0.85
V$PERO	0.75	0.87	1	0.67	1.21
V$PIT1	1.08	0.33	0.58	0.78	0.65
V$PLAG	0.98	1.88	1.22	1.7	1.38
V$PLZF	1.27	0.46	1.59	0.76	0.89
V$PPAR	0.81	0.87	0.76	0.62	0.85
V$PRDF	0.92	0.57	1.21	0.8	1.01
V$PTF1	1.13	1.21	0.75	0.79	0.99
V$PURA	0.96	2.08	1.14	0.98	0.54
V$RBIT	0.4	0.21	1.3	0.61	1.11
V$RBP2	1.38	0	0.98	1	0.83
V$RBPF	1.34	1.36	1.23	0.88	0.77
V$RORA	0.92	0.52	1.05	0.74	0.95
V$RP58	0.58	1.24	1.14	1.27	1.08
V$RREB	1.22	1.9	0.63	1.21	1.05
V$RU49	0.99	0.3	1.1	1.15	0.79
V$RUSH	0.85	1.3	1.08	0.61	0.99
V$RXRF	1.1	0.76	1.05	0.84	1.16
V$SATB	0.72	0.39	1.09	0.82	0.75
V$SF1F	0.69	1.48	1.37	0.65	1.51
V$SMAD	0.74	1.42	0.92	1.05	1.04
V$SNAP	1.02	0.35	1.02	0.8	1.28
V$SORY	1.01	0.93	1.11	1.01	1.1
V$SP1F	1.03	2.04	1.17	1.38	1.26
V$SPZ1	0.97	1.14	0.91	1.26	0.74
V$SREB	1.59	1.47	0.96	1.04	1.11
V$SRFF	1.49	1.56	1.15	1.04	1.19
V$STAF	0.98	0.75	1.03	1	0.81
V$STAT	1.33	1.37	0.71	1.01	1.15
V$TALE	0.74	0.79	1.01	0.86	1.03
V$TCFF	0.37	1.47	1.03	0.38	1.55
V$TEAF	1.53	1	0.85	1.08	0.95
V$THAP	0.47	1.57	0.72	1.07	0.65
V$WHNF	0.42	0	0.87	1.59	0.44
V$XBBF	0.74	1.7	0.66	1.37	0.71
V$YBXF	0.55	1.49	1.42	1.06	1.36
V$YY1F	0.63	1.2	1.06	0.77	1.22
V$ZBPF	0.97	1.91	1.2	1.49	1.09
V$ZF35	0.72	1.55	0.76	1.32	1.61
V$ZF5F	0.62	0.36	1.45	2.01	0.71
V$ZFHX	0.85	0.71	0.83	0.77	0.99
V$ZFIA	1.09	0.94	0.95	1.11	1.22
V$ZNFP	1.03	1.38	1.08	1.31	1.26
