# Supplementary material for: Overrepresentation of transcription factor families in the genesets underlying breast cancer subtypes
Source: BMC Genomics. 2012 May 22;13:199. doi: 10.1186/1471-2164-13-199 (PMC3441847; doi:10.1186/1471-2164-13-199)
Supplement: Additional file 11 — Table S4. Correlation between TFBS overrepresentation and mRNA expression of corresponding TF genes. Table displays the Pearson’s correlation between the geometric mean of expression values of transcription factor genes in subtypes and fold overrepresentation of corresponding TFBS families. [file 1471-2164-13-199-S11.pdf]

*Supplementary Table 4*: Table displays the pearson´s correlation between the geometric mean of expression values of transcription factor genes in subtypes and fold overrepresentation of corresponding TFBS familes

| Gene Symbol | GeneName | TFBS families | Pearson's correlation |
|---|---|---|---|
| SNFT | Jun dimerization protein p21SNFT | V$AP1F | 0.37 |
| FOS | v-fos FBJ murine osteosarcoma viral oncogene homolog | V$AP1F | 0.65 |
| FOSB | FBJ murine osteosarcoma viral oncogene homolog B | V$AP1F | 0.83 |
| FOSL2 | FOS-like antigen 2 | V$AP1F | 0.85 |
| FOSL1 | FOS-like antigen 1 | V$AP1F | 0.43 |
| JDP2 | jun dimerization protein 2 | V$AP1F | 0.48 |
| JUN | jun oncogene | V$AP1F | 0.74 |
| JUNB | jun B proto-oncogene | V$AP1F | 0.72 |
| JUNB | jun B proto-oncogene | V$AP1F | 0.66 |
| ARID5B | AT rich interactive domain 5B (MRF1-like) | V$ARID | -0.16 |
| ARID5B | AT rich interactive domain 5B (MRF1-like) | V$ARID | -0.07 |
| ARID5B | AT rich interactive domain 5B (MRF1-like) | V$ARID | 0.27 |
| CUTL1 | cut-like 1, CCAAT displacement protein (Drosophila) | V$CLOX | 0.14 |
| CUTL2 | cut-like 2 (Drosophila) | V$CLOX | -0.74 |
| E2F1 | E2F transcription factor 1 | V$E2FF | 0.34 |
| E2F2 | E2F transcription factor 2 | V$E2FF | 0.15 |
| E2F3 | E2F transcription factor 3 | V$E2FF | -0.21 |
| E2F4 | E2F transcription factor 4, p107/p130-binding | V$E2FF | -0.53 |
| E2F4 | E2F transcription factor 4, p107/p130-binding | V$E2FF | -0.68 |
| E2F5 | E2F transcription factor 5, p130-binding | V$E2FF | 0.81 |
| E2F7 | E2F transcription factor 7 | V$E2FF | 0.37 |
| E2F8 | E2F transcription factor 8 | V$E2FF | 0.18 |
| TFDP1 | transcription factor Dp-1 | V$E2FF | -0.01 |
| TFDP1 | transcription factor Dp-1 | V$E2FF | -0.05 |
| ATF6 | activating transcription factor 6 | V$EBOX | 0.88 |
| CREBL1 | cAMP responsive element binding protein-like 1 | V$EBOX | 0.49 |
| MAX | MYC associated factor X | V$EBOX | 0.85 |
| MGA | MAX gene associated | V$EBOX | -0.58 |
| MLX | MAX-like protein X | V$EBOX | 0.26 |
| MLXIPL | MLX interacting protein-like | V$EBOX | 0.78 |
| MLXIPL | MLX interacting protein-like | V$EBOX | -0.83 |
| MLXIPL | MLX interacting protein-like | V$EBOX | 0.76 |
| MYC | v-myc myelocytomatosis viral oncogene homolog (avian) | V$EBOX | -0.38 |
| MYCN | v-myc myelocytomatosis viral related oncogene, neuroblastoma derived (avian) | V$EBOX | -0.35 |
| TCF4 | transcription factor 4 | V$EBOX | -0.36 |
| EGR1 | early growth response 1 | V$EGRF | -0.76 |
| EGR2 | early growth response 2 (Krox-20 homolog, Drosophila) | V$EGRF | -0.78 |
| EGR3 | early growth response 3 | V$EGRF | -0.51 |
| ZBTB7A | zinc finger and BTB domain containing 7A | V$EGRF | 0.29 |
| ZBTB7B | zinc finger and BTB domain containing 7B | V$EGRF | -0.02 |
| KLF2 | Kruppel-like factor 2 (lung) | V$EKLF | -0.01 |
| KLF2 | Kruppel-like factor 2 (lung) | V$EKLF | -0.21 |
| KLF3 | Kruppel-like factor 3 (basic) | V$EKLF | -0.16 |
| KLF4 | Kruppel-like factor 4 (gut) | V$EKLF | -0.13 |
| KLF6 | Kruppel-like factor 6 | V$EKLF | -0.34 |
| KLF6 | Kruppel-like factor 6 | V$EKLF | -0.75 |
| KLF6 | Kruppel-like factor 6 | V$EKLF | -0.21 |
| KLF7 | Kruppel-like factor 7 (ubiquitous) | V$EKLF | 0.86 |
| KLF8 | Kruppel-like factor 8 | V$EKLF | 0.09 |
| KLF8 | Kruppel-like factor 8 | V$EKLF | -0.21 |
| TRPS1 | trichorhinophalangeal syndrome I | V$GATA | 0.37 |
| GATA2 | GATA binding protein 2 | V$GATA | -0.04 |
| GATA3 | GATA binding protein 3 | V$GATA | 0.35 |
| GATA6 | GATA binding protein 6 | V$GATA | -0.67 |
| GATAD2A | GATA zinc finger domain containing 2A | V$GATA | -0.31 |
| GATAD1 | GATA zinc finger domain containing 1 | V$GATA | 0.61 |
| GATAD1 | GATA zinc finger domain containing 1 | V$GATA | 0.50 |
| GLI1 | glioma-associated oncogene homolog 1 (zinc finger protein) | V$GLIF | -0.87 |
| GLI2 | GLI-Kruppel family member GLI2 | V$GLIF | -0.84 |
| GLI2 | GLI-Kruppel family member GLI2 | V$GLIF | -0.79 |
| GLI3 | GLI-Kruppel family member GLI3 (Greig cephalopolysyndactyly syndrome) | V$GLIF | -0.10 |
| GLIS1 | GLIS family zinc finger 1 | V$GLIF | -0.69 |
| GLIS2 | GLIS family zinc finger 2 | V$GLIF | 0.17 |
| ZIC1 | Zic family member 1 (odd-paired homolog, Drosophila) | V$GLIF | -0.18 |
| ZIC1 | Zic family member 1 (odd-paired homolog, Drosophila) | V$GLIF | -0.22 |
| ZIC4 | Zic family member 4 | V$GLIF | -0.02 |
| ZIC5 | Zic family member 5 (odd-paired homolog, Drosophila) | V$GLIF | -0.22 |
| AR | androgen receptor (dihydrotestosterone receptor; testicular feminization; spinal and bulbar muscular atrophy; Kennedy disease) | V$GREF | -0.09 |
| NR3C1 | nuclear receptor subfamily 3, group C, member 1 (glucocorticoid receptor) | V$GREF | -0.48 |

| Gene Symbol | GeneName | TFBS families | Pearson's correlation |
|---|---|---|---|
| NR3C2 | nuclear receptor subfamily 3, group C, member 2 | V$GREF | 0.12 |
| PGR | progesterone receptor | V$GREF | -0.61 |
| CBFA2T2 | core-binding factor, runt domain, alpha subunit 2; translocated to, 2 | V$HAML | 0.38 |
| CBFA2T3 | core-binding factor, runt domain, alpha subunit 2; translocated to, 3 | V$HAML | -0.11 |
| CBFB | core-binding factor, beta subunit | V$HAML | 0.48 |
| CBFB | core-binding factor, beta subunit | V$HAML | 0.43 |
| RUNX1 | runt-related transcription factor 1 (acute myeloid leukemia 1; aml1 oncogene) | V$HAML | -0.37 |
| RUNX1T1 | runt-related transcription factor 1; translocated to, 1 (cyclin D-related) | V$HAML | -0.56 |
| RUNX2 | runt-related transcription factor 2 | V$HAML | -0.07 |
| RUNX3 | runt-related transcription factor 3 | V$HAML | 0.06 |
| HOXA2 | homeobox A2 | V$HOXF | -0.34 |
| HOXA3 | homeobox A3 | V$HOXF | -0.47 |
| HOXA4 | homeobox A4 | V$HOXF | -0.34 |
| HOXA5 | homeobox A5 | V$HOXF | -0.34 |
| HOXB2 | homeobox B2 | V$HOXF | 0.27 |
| HOXB2 | homeobox B2 | V$HOXF | 0.33 |
| HOXB3 | homeobox B3 | V$HOXF | 0.17 |
| HOXB3 | homeobox B3 | V$HOXF | 0.08 |
| HOXB4 | homeobox B4 | V$HOXF | -0.11 |
| HOXB4 | homeobox B4 | V$HOXF | -0.70 |
| HOXB5 | homeobox B5 | V$HOXF | 0.06 |
| HOXB6 | homeobox B6 | V$HOXF | 0.08 |
| HOXB6 | homeobox B6 | V$HOXF | 0.10 |
| HOXB7 | homeobox B7 | V$HOXF | -0.22 |
| HOXB8 | homeobox B8 | V$HOXF | 0.37 |
| HOXC5 | homeobox C5 | V$HOXF | 0.16 |
| HOXD8 | homeobox D8 | V$HOXF | -0.13 |
| HOXA7 | homeobox A7 | V$HOXF | -0.32 |
| IRF1 | interferon regulatory factor 1 | V$IRFF | -0.40 |
| IRF4 | interferon regulatory factor 4 | V$IRFF | -0.98 |
| IRF4 | interferon regulatory factor 4 | V$IRFF | -0.88 |
| IRF8 | interferon regulatory factor 8 | V$IRFF | -0.98 |
| LHX2 | LIM homeobox 2 | V$LHXF | -0.69 |
| LHX6 | LIM homeobox 6 | V$LHXF | -0.24 |
| LMX1B | LIM homeobox transcription factor 1, beta | V$LHXF | 0.68 |
| MAZ | MYC-associated zinc finger protein (purine-binding transcription factor) | V$MAZF | 0.15 |
| MAZ | MYC-associated zinc finger protein (purine-binding transcription factor) | V$MAZF | 0.03 |
| PATZ1 | POZ (BTB) and AT hook containing zinc finger 1 | V$MAZF | 0.49 |
| PATZ1 | POZ (BTB) and AT hook containing zinc finger 1 | V$MAZF | 0.55 |
| PATZ1 | POZ (BTB) and AT hook containing zinc finger 1 | V$MAZF | 0.58 |
| SIX5 | sine oculis homeobox homolog 5 (Drosophila) | V$MEF3 | -0.39 |
| ZNF239 | zinc finger protein 239 | V$MOKF | -0.54 |
| MYT1 | myelin transcription factor 1 | V$MYT1 | 0.34 |
| MZF1 | myeloid zinc finger 1 | V$MZF1 | -0.48 |
| HIVEP1 | human immunodeficiency virus type I enhancer binding protein 1 | V$NFKB | 0.21 |
| HIVEP2 | human immunodeficiency virus type I enhancer binding protein 2 | V$NFKB | 0.38 |
| HIVEP3 | human immunodeficiency virus type I enhancer binding protein 3 | V$NFKB | -0.75 |
| NFKB2 | nuclear factor of kappa light polypeptide gene enhancer in B-cells 2 (p49/p100) | V$NFKB | 0.08 |
| REL | v-rel reticuloendotheliosis viral oncogene homolog (avian) | V$NFKB | -0.58 |
| RELB | v-rel reticuloendotheliosis viral oncogene homolog B, nuclear factor of kappa light polypeptide gene enhancer in B-cells 3 (avian) | V$NFKB | -0.18 |
| ZNF423 | zinc finger protein 423 | V$OAZF | -0.16 |
